# Baa-iov: Blockchain-enabled anonymous authentication for internet of vehicles

**DOI:** 10.1371/journal.pone.0347787

**Published:** 2026-04-24

**Authors:** Wenfeng Huang, Si Chen

**Affiliations:** 1 School of Ocean Engineering, Xiamen Ocean Vocational College, Xiamen, China; 2 School of Information Engineering, Xiamen Ocean Vocational College, Xiamen, China; University of Electronic Science and Technology of China, CHINA

## Abstract

As a core component of intelligent transportation systems, the Internet of Vehicles (IoV) relies heavily on secure and efficient authentication mechanisms to support real-time information exchange and data sharing among network entities. However, traditional centralized authentication schemes widely adopted in current IoV systems suffer from several drawbacks, including performance bottlenecks, inefficient cross-domain authentication, and insufficient identity privacy protection, which severely restrict the security and scalable deployment of IoV systems. To address these challenges, this paper proposes a novel blockchain-based multi-Trusted Authority (TA) collaborative authentication and key agreement scheme. The scheme adopts a decentralized cross-TA authentication architecture based on blockchain-enabled trust transfer to eliminate single point of failure risks. Furthermore, it constructs an anonymous authentication protocol supporting both vehicle-to-TA and vehicle-to-vehicle key agreement, ensuring identity privacy and resisting various security attacks. Comprehensive security validation, including Burrows-Abadi-Needham (BAN) logic formal analysis, informal security verification, and ProVerif-based verification, confirms the scheme’s strong security guarantees. Comparative evaluations with existing schemes demonstrate that the proposed scheme achieves reasonable energy consumption while ensuring high security performance. Specifically, its computational cost is reduced by 39.42% compared with the best existing scheme.

## 1 Introduction

In the Internet of Vehicles (IoV), most existing authentication protocols are based on a single Trusted Authority (TA) model and assume that the TA has unlimited resources. However, in smart city scenarios involving multi-domain collaboration, a single TA model is no longer sufficient. Therefore, this study focuses on designing secure and efficient authentication and key agreement mechanisms in a high-speed IoV environment with multi-domain management.

The main technical challenges and corresponding design choices of this study are as follows: 1) Balancing Security and Efficiency in Cross-TA Authentication [1]. This challenge can be addressed through the decentralized trust mechanism of blockchain. The high mobility of vehicles may lead to frequent cross-domain transitions (e.g., moving from the TA management domain of City A to that of City B). Cross-domain vehicles need to complete cross-TA identity verification, but there is a trade-off between security and efficiency in current multi-TA authentication systems. If a domain-by-domain secondary authentication mechanism is adopted, it will cause traffic delays due to lengthy procedures, which conflicts with the high-speed interaction requirements of the IoV; if the authentication process is simplified, it may lead to security risks arising from differences in security policies. The distributed ledger feature of blockchain can effectively solve the above problems: it enables trusted authentication and sharing of identity information across TA domains, allowing verification to be completed without repeatedly submitting credentials. Additionally, the immutable nature of blockchain can prevent identity forgery. This is the key reason for choosing blockchain as the foundation for cross-TA trust, as it can balance security and efficiency simultaneously. 2) Resource Consumption Caused by Roadside Unit (RSU) Function Overload. This challenge can be addressed through function optimization combined with lightweight communication protocols. Existing solutions design RSUs as dual-function nodes (i.e., relay and authentication), which are required to forward data and participate in core operations such as hash computation and signature verification. However, RSUs have limited hardware resources (e.g., computing power and storage capacity). In high-density scenarios (e.g., peak-hour urban roads), this may lead to computing power saturation and bandwidth waste, potentially causing network performance degradation. Therefore, it is necessary to optimize the functions of RSUs: remove their authentication capabilities, retain only their relay role, and assign the authentication function to TAs, which have more abundant resources. The underlying logic of this design is to match hardware resources with functional responsibilities. Meanwhile, the solution integrates lightweight message transmission protocols to further reduce the communication overhead of RSUs, which aligns with the low-latency requirements of the IoV. 3) Security Risk Accumulation Caused by High-Frequency Interactions Between RSUs and TAs. This challenge can be mitigated by combining Elliptic Curve Cryptography (ECC) with anonymous authentication mechanisms. Each authentication process requires at least two to three message exchanges between an RSU and a TA. High-frequency interactions not only increase system overhead but also make the system more vulnerable to attacks and key compromise. ECC ensures the security of data transmission through public-key cryptographic mechanisms. At the same time, anonymous authentication technology allows vehicles to hide their real identities during authentication and only submit anonymous credentials to TAs. The core goal of this technology selection is to reduce the attack surface: it not only resists data tampering and theft through encryption but also reduces the risk of identity information abuse through anonymity.

To address the aforementioned issues, this paper proposes a blockchain-based multi-TA collaborative authentication and key agreement scheme. This solution establishes a decentralized trust architecture leveraging blockchain technology, enabling secure collaboration and trust transfer between TAs. The TA acts as the primary authentication entity, while the RSU is only responsible for relaying authentication messages. Furthermore, the protocol adopts anonymous authentication to ensure vehicle identity privacy and security, as well as secure key agreement between vehicles and TAs, and among vehicles themselves. The proposed solution improves system performance and scalability while maintaining strong security.

### 1.1 Key contributions and innovations

This study addresses core challenges in IoV identity authentication and key agreement by proposing an innovative blockchain-based solution. Compared to existing research, its primary contributions and innovations are as follows:

1) Innovative multi-TA collaborative authentication architecture

Overcoming the limitations of traditional single-trusted-authority models, this research proposes a blockchain-based multi-trusted-authority collaborative authentication framework. By enabling trusted data sharing among authorities via smart contracts, it resolves cross-domain authentication challenges while significantly enhancing system scalability.

2) Multi-layer security protection mechanism

Building upon conventional hash and XOR operations, it incorporates elliptic curve point multiplication and designs a dynamic blockchain pointer encryption mechanism, constructing a more robust security system resistant to various known attacks.

3) Optimized network resource allocation strategy

Redefines the functional boundaries of roadside units, limiting them to data relay nodes while delegating core authentication computation tasks to trusted authorities. This architectural innovation reduces network load while minimizing potential security exposure.

4) Efficient Vehicle-to-Vehicle key agreement protocol

Proposes a direct vehicle-to-vehicle key agreement mechanism based on blockchain-stored shared parameters. This significantly enhances authentication efficiency while maintaining high security, making it particularly suitable for high-density IoV environments.

5) Comprehensive Security and Performance Validation

The solution undergoes multidimensional verification through formal BAN logic analysis, informal security arguments, and ProVerif simulation. By optimizing parameter storage and simplifying computational workflows, security is substantially enhanced without compromising computational or communication efficiency. Experimental results demonstrate acceptable overall performance compared to existing mainstream solutions.

### 1.2 Paper organization

The remainder of this paper is organized as follows: Section 2 introduces the fundamental concepts required for understanding the proposed scheme, including blockchain, ECC, and BAN logic. Section 3 systematically analyzes the limitations of existing authentication protocols and blockchain-based solutions, while identifying research gaps. Section 4 formally defines the system model, threat model, security objectives, and security measures. Section 5 elaborates on the blockchain-based multi-TA collaborative authentication mechanism, comprising three phases: system initialization, entity registration, mutual authentication and key agreement. Section 6 rigorously evaluates the security of the proposed scheme through BAN logic verification, informal security analysis, and formal validation using the ProVerif tool. Section 7 presents a comprehensive performance comparison with five state-of-the-art protocols in terms of computational and communication overhead. Finally, Section 8 summarizes the key contributions and discusses potential future research directions in IoV security.

## 2 Preliminaries

### 2.1 Blockchain

The fundamental principles of blockchain comprise distributed storage, consensus mechanisms, and cryptographic algorithms. Distributed storage involves replicating and storing data across the network, while consensus mechanisms ensure data consistency among network nodes. Cryptographic algorithms are employed to safeguard the confidentiality and integrity of data. These basic principles collectively form the foundational architecture of blockchain, enabling decentralization while ensuring the security and reliability of data.

### 2.2 ECC

ECC is a public-key cryptographic system based on the mathematical structure of elliptic curves. Users can establish a secure elliptic curve by appropriately choosing parameters. The encryption and signature processes involve mathematical operations on points on the elliptic curve, including point addition, doubling, and other operations. Compared to traditional asymmetric encryption algorithms like RSA, ECC offers higher security with smaller key lengths, making it more suitable for resource-limited environments. The following subsections present the mathematical foundations and algorithms underlying ECC.

1) Elliptic Curve Equation

$$E:y^{\text{2}}=x^{\text{3}}+ax+b(\text{mod }p)$$

Parameters:

*p*: Large prime defining finite field F_*p*_

*a,b*: Curve coefficients (*4a*^*3*^*+27b*2  ≡ *0*(mod *p*))

#### G

: Basepoint (generator) of prime order *n*

2) Elliptic Curve Diffie-Hellman (ECDH)

Key Generation:

Alice: Private key *d*_*A*_$\overset{R}{\leftarrow }$[1, n−1], Public key *Q*_*A*_*=d*_*A*_*G*

Bob: Private key *d*_*B*_$\overset{R}{\leftarrow }$[1,n−1], Public key *Q*_*A*_*=d*_*B*_*G*

Shared Secret Calculation:


$$K=d_{A}Q_{B}=d_{B}Q_{A}=\left(d_{A}d_{B}\right)G$$

Security: Relies on ECDH assumption (hard to compute *d*_*A*_*d*_*B*_*G* given *Q*_*A*_, *Q*_*B*_)

3) Elliptic Curve Digital Signature Algorithm (ECDSA)

Signature Generation (for message *m*):

Compute hash *h = H*(*m*), interpreted as integer in Z_n_, Select random *k*$\overset{R}{\leftarrow }$[*1, n − 1*], Compute (*x,y*)*=kG*, Output signature (*r, s*): *r = x mod n, s = k*^*−1*^(*h + d*_*A*_*r*) *mod n*.

Signature Verification:

Verify *r, s*∈[*1,n − 1*], Compute *h = H(m)*h, Calculate: *u*_*1*_ *≡ hs*^*−1*^(mod *n*)*, u*_*2*_ *≡ rs*^*−1*^(mod *n*)*,* (*x,y*)*=u*_*1*_*G+u*_*2*_*Q*_*A*_.

Accept if *r ≡ x* (mod *n*).

### 2.3 BAN logic

BAN logic, proposed by Burrows, Abadi, and Needham in 1989, is a formal approach for analyzing and verifying cryptographic protocols. It translates protocol messages into logical formulas and applies inference rules to derive the beliefs and knowledge of the participating entities. By enabling structured analysis, BAN logic aids in identifying and addressing vulnerabilities to bolster the overall robustness of cryptographic systems.

## 3 Related works

In light of the research direction of this study, the relevant literature is categorized into two parts: the authentication schemes for in-vehicle self-organizing networks and blockchain applications in IoV.

### 3.1 Authentication schemes for in-vehicle self-organizing networks

1) Conditional Privacy-Preserving Authentication with Efficient Revocation Mechanisms

This category includes schemes that achieve conditional privacy while optimizing communication and computational efficiency via novel revocation mechanisms. Zhong et al.[2] proposed a conditional privacy-preserving authentication scheme based on registration lists, which reduces communication overhead by substituting traditional revocation lists and mitigates malicious vehicle behavior. Alazzawi et al.[3] proposed a novel pseudo-identity-based solution for achieving conditional anonymity in Vehicular Ad Hoc Networks (VANETs), which discards bilinear pairings and certificate revocation lists, thus significantly improving the efficiency of signing and authentication. However, Al-Shareeda et al.[4] identified that the scheme of Alazzawi et al. suffers from vulnerabilities in privacy preservation and unlinkability, fails to provide password modification functionality, and is susceptible to side-channel attacks. To address these issues, Al-Shareeda et al. redesigned a conditional privacy-preserving authentication scheme based on elliptic curve cryptography, effectively resolving the aforementioned problems.

Overall, existing conditional privacy-preserving schemes still have room for improvement in achieving efficient revocation and privacy trade-offs, particularly lacking lightweight solutions that simultaneously satisfy low communication overhead, strong privacy protection, and resistance to physical side-channel attacks.

2) Certificateless and lightweight authentication architectures

These schemes eliminate traditional certificate management through certificateless or identity-based cryptographic constructs while maintaining low computational overhead. Cui et al.[5] proposed an extensible authentication mechanism for multi-cloud environments, leveraging ECC and simplifying service selection complexity through cloud brokers to meet diversified service demands. Liu et al.[6] proposed an authentication scheme integrating anonymous identity generation, trust authority verification, and a reputation evaluation mechanism to dynamically assess vehicle credibility based on historical interaction behavior. Ma et al.[7] proposed a scheme that does not rely on bilinear pairings, supporting mutual authentication and generates secure session keys for secret communication while preserving privacy. Wu et al.[8] introduced a certificateless aggregated signatures scheme for IoV to address the problem of frequent key updates by vehicles. Tangade et al.[9] proposed an identity-based scheme utilizing keyed-Hash Message Authentication Code (HMAC) that employs reward points to calculate vehicle trust values. However, it does not support batch verification of messages and signatures. Su et al.[10] proposed a scheme for Vehicle-to-Grid (V2G) networks employing non-supersingular elliptic curves.

Although certificateless and lightweight architectures eliminate complex certificate management, most solutions still face the following challenges: difficulty in efficiently supporting batch verification across large-scale vehicular networks, communication overhead caused by frequent key updates, and ensuring equivalent security levels without introducing bilinear pairing operations.

3) Multi-factor and physically enhanced authentication schemes

This category encompasses methods that incorporate Physical Unclonable Functions (PUF), biometrics, or multi-factor protocols to enhance security and resilience against physical attacks. Li et al.[11] proposed a scheme based on anonymous identity, introducing a new authentication architecture and hiding the computation of the user’s identity through a one-way hash function to enhance privacy and security. Alfadhli et al.[12] proposed a scheme combining PUFs and one-time dynamic pseudo-identities as factors for authentication to improve overall security and efficiency. Xu et al.[13] proposed a scheme that delineates distinct roles within system operations. During the initialization phase, the System Administrator (SA) performs security registration in a specified environment. Subsequently, the TA takes responsibility for disseminating authentication parameters to the RSUs. Umar et al.[14] proposed a PUF-based scheme for secure and efficient data transfer over public channels, though its anonymity relies on trusted third parties for pseudonym updates. Jiang et al.[15] proposed a PUF-based scheme integrating PUFs, passwords, and biometrics to achieve multifactor authentication, thus increasing the difficulty of forgery by adversaries.

While incorporating physical factors such as PUFs or biometrics enhances security, it also introduces new challenges, including the instability of PUF responses, privacy concerns for biometric templates, and increased user experience complexity and deployment costs associated with multi-factor protocols.

4) Advanced cryptographic protocols and group-oriented authentication

These approaches employ advanced cryptographic techniques such as chaotic maps, puncturable signatures, and group authentication to achieve scalability, forward secrecy, and efficient batch verification. Wei et al.[16] proposed a bidirectional anonymous traceable group authentication scheme for IoV. Their scheme manages multiple RSUs through fast dynamic grouping and uses the unidirectional trapdoor nature and semigroup property of Chebyshev chaotic mapping to authenticate the access of vehicles entering the RSU groups. Hou et al.[17] introduced a lightweight PUF into the authentication scheme and integrated the in-vehicle unit with a 5G Subscriber Identity Module (SIM) card to address identity forgery issues. Sripathi et al.[18] proposed a scheme for nonlinear pairings that supports batch verification by using a certificate-less signature mechanism to avoid certificate management and key escrow issues. Xie et al.[19] employed PUFs to resist RSU capture attacks while enhancing security through a three-factor secrecy strategy. Hou et al.[20] proposed an efficient two-factor authentication protocol by integrating blockchain with Trusted Execution Environment (TEE) technology, which not only guarantees anonymity but also reduces computational overhead. Jiang et al.[21] developed a two-level security framework based on blockchain and ensemble learning, achieving lightweight authentication via PUF while utilizing Whale Optimization Algorithm (WOA) and Extreme Gradient Boosting (XGBoost) for malicious attack detection. Xiong et al.[22] introduced a mutual authentication protocol supporting fine-grained forward/backward security through the combination of puncturable signatures and parallel key-insulated proxy re-signature schemes. Bhatt et al.[23] proposed a blockchain-based conditional privacy-preserving authentication scheme for temporary platoon communications, reducing RSU reliance and infrastructure costs while incorporating an accident prevention mechanism. Ibrahim et al.[24] proposed the PPA6-IoV protocol featuring a six-step authentication process to preserve vehicle privacy while effectively reducing both communication and computational costs. Zhang et al.[25] proposed a system based on a trusted connection architecture, which introduced a platform authentication mechanism and effectively improved communication security in IoV environments.

Group authentication and advanced cryptographic protocols—such as chaotic mapping and puncturable signatures—demonstrate advantages in scalability and forward secrecy. However, their computational complexity is typically high, and most schemes fail to achieve efficient group dynamic management within highly dynamic vehicular ad hoc networks.

5) Decentralized IoV authentication

Recent research emphasizes the shift toward decentralized authentication in the IoV to address scalability, security, and privacy challenges. The edge enhancement protocol proposed by Lo et al.[26] reduces cloud dependencies and improves system resilience by leveraging a consortium blockchain. The BAKARI (Blockchain-Powered AKA Scheme with a Reputation-Centric) proposed by Mukathe et al.[27] is a blockchain-based key agreement scheme with reputation incentives. Haider et al.[28] proposed the Blockchain Enabled Secure Authentication Protocol for IoVs (BESA-IOV), which utilizes ECC and blockchain for a lightweight authentication mechanism to achieve the goal of reducing latency and cost. Borges et al.[29] proposed using decentralized reputation management to verify identity, and this approach has the advantage of ensuring identity security even in disconnected environments. Wang et al.[30] introduced BCADS, a blockchain-assisted cross-domain authentication scheme with decentralized identity for VANETs under strict oversight, enabling autonomous identifier management and reducing computational and communication costs by 36.6% and 87.1%. Liu et al.[31] proposed a Cybertwin-enabled distributed authentication scheme that employs lightweight cryptography and digital twins for real-time behavior tracking and dynamic trust distribution, reducing authentication latency in high-density vehicular environments.

Emerging decentralized solutions leverage technologies such as blockchain to mitigate single points of failure and trust issues. However, they still face widespread performance bottlenecks, including high transaction latency and substantial consensus overhead, and lack effective mechanisms to balance computational and communication loads between resource-constrained in-vehicle units and powerful backend systems.

### 3.2 Blockchain-related research in the field of IoV

1) Authentication and Key Management Schemes

This category focuses on leveraging blockchain’s decentralization, immutability, and traceability to construct novel authentication frameworks and key management systems. Dorri et al.[32] presented an innovative lightweight and scalable blockchain system designed to meet the high demands of the IoV. However, their approach relies on centralized key management lists that are susceptible to compromise, leading to large-scale key compromise incidents. To address these challenges, Wang et al.[33] proposed a scheme for IoV that fully integrates blockchain technology, including consensus-mechanism-driven smart contracts and cryptographic accumulator-based Public Key Infrastructure (PKI) mechanisms. Zhang et al.[34] proposed a blockchain-based asymmetric group key agreement protocol for IoV, aiming to protect user privacy through anonymous authentication techniques. Qureshi et al.[35] proposed an efficient, secure, and anonymous blockchain-based conditional privacy protection and authentication mechanism. They implemented a blockchain using Hyperledger Fabric to enable vehicle nodes to share data anonymously and maintain anonymity, traceability, and unlinkability during data communication. Ma et al.[36] proposed an innovative decentralized key management scheme in the field of VANETs, with the main goal of achieving automatic registration, updating, and revocation of users’ public keys to ensure communication security. However, this scheme may introduce high computational complexity for resource-constrained devices in VANETs. Lin et al.[37] employed blockchain technology to solve key update and certificate management challenges in public key cryptographic authentication systems. They combined blockchain technology with key derivation algorithms to proactively manage certificates for secure authentication. Li et al.[38] proposed a blockchain-assisted revocable cross-domain authentication scheme for VANETs, supporting different authentication methods across domains and enabling malicious vehicle revocation via group public key updates while avoiding blockchain-induced latency. Akhter et al.[39] designed a blockchain-based switching authentication scheme to reduce the redundant computational overhead incurred by vehicles when switching RSUs. Singh et al.[40] proposed a lightweight group-based authentication protocol for 5G-enabled IoV networks, achieving mutual authentication, forward/backward secrecy, and session unlinkability. AVISPA and BAN logic validation confirm attack resistance, with signaling overhead reduced by 47.3% and bandwidth consumption by 32.3%.

Existing blockchain-based authentication and key management solutions effectively enhance traceability and tamper resistance. However, their performance is often constrained by the inherent throughput and latency limitations of blockchain technology. Moreover, designing truly lightweight on-chain operations suitable for vehicle-embedded environments remains an open challenge.

2) Certificateless and lightweight cryptographic schemes

These schemes eliminate traditional certificate management through lightweight cryptographic constructs and efficient algorithms. Ali et al.[41] proposed an innovative certificateless public key signature solution utilizing bilinear pairing techniques aimed at enhancing the privacy security of vehicle-to-infrastructure communications. However, this solution utilizes bilinear pairing for batch signature aggregation and verification, which leads to the challenge of high computational complexity. Meng et al.[42] proposed a lightweight anonymous mutual authentication and key agreement scheme, enabling efficient cross-regional node authentication and session key establishment. Tan et al.[43] introduced a VANET system model incorporating edge computing infrastructure, which provides sufficient computational and storage resources for individual vehicles. Vishwakarma et al.[44] proposed a lightweight blockchain security scheme supporting Software-Defined Networking (SDN) for secure storage and communication, enabling vehicles to generate their own key pairs and obtain temporary credentials during registration.

Despite adopting certificateless and lightweight designs, many solutions struggle to balance robust privacy protection (such as identity unlinkability) with efficient cross-domain authentication while achieving fully decentralized trust.

3) Privacy-preserving and conditional anonymity schemes

This category comprises identity protection, conditional traceability, and communication privacy without sacrificing security. Bhushan et al.[45] proposed a vehicular blockchain network model introducing a consensus authentication model to smart city infrastructure, handling security and traffic control via specialized miner nodes but heavily relying on sensor data and underutilizing smart contracts and PKI technologies. Feng et al.[46] proposed an Efficient Privacy-Preserving Authentication Model (EPAM) using asynchronous accumulators to extend blockchain applications for efficient membership verification and pseudonym management, though its three-level model may add computational overhead. Xu et al.[47] proposed a blockchain and token-based scheme reducing costs through time-sensitive tokens but is vulnerable to Man-In-The-Middle (MITM) attacks. Son et al.[48] proposed a blockchain-based Vehicle-to-Infrastructure (V2I) authentication scheme comprising five phases. The initial authentication phase uses ECC for security authentication, and authentication data is stored on the blockchain. If the RSU discovers vehicle misbehavior, it can execute vehicle revocation through the blockchain. Lu et al.[49] proposed a lattice-based dual blockchain anonymous authentication scheme featuring forward security and revocability for VANETs. The dual-chain architecture decouples identity from mobility to enhance anonymity. Bonsai tree structures ensure forward security, and malicious vehicles can be anonymously revoked. This work addresses quantum computing threats while maintaining balanced security and efficiency.

Existing privacy-preserving solutions strike a good balance between conditional anonymity and traceability, but they often rely on complex cryptographic accumulators or multi-layer architectures. This can impose significant storage and computational burdens in practical deployments and lack sufficient support for rapid revocation of dynamic vehicle members.

4) Hybrid and multi-technology integrated schemes

These approaches combine blockchain with other technologies such as edge computing, software-defined networking (SDN), and advanced consensus mechanisms to achieve scalable and efficient authentication. Mei et al.[50] proposed a scheme that combines blockchain, IoV, and edge computing to support efficient computation and storage functions. Xu et al.[51] proposed a scheme for IoV that utilizes blockchain and RSU-assisted technologies, aiming to address the complex authentication problems faced by vehicles in the transportation domain. Shi et al.[52] proposed a blockchain-enabled domain name service and mutual authentication protocol based on the Blockchain-enabled Authentication and Communications Network (BeACONS) framework, significantly reducing reliance on centralized infrastructure for inter-vehicle communications. Surapaneni et al.[53] developed a handover authentication scheme combining blockchain with the InterPlanetary File System (IPFS), utilizing a Proof-of-Reputation (PoR) consensus mechanism to substantially improve vehicle re-authentication efficiency. Lin et al.[54] enhanced their protocol by employing blockchain to implement distributed trusted third-party services, effectively preventing single points of failure. The BAKARI scheme proposed by Mukathe et al.[27] achieves secure key agreement through Schnorr signatures and elliptic curve cryptography while employing blockchain smart contracts for vehicle reputation management. Ma et al.[55] introduced an optimized Practical Byzantine Fault Tolerance (PBFT) consensus-based distributed authentication scheme, where smart-contract-automated authentication processes enable the reuse of authentication results.

Integrating blockchain with technologies such as edge computing and SDN represents a cutting-edge research direction. However, current research still faces significant gaps in designing collaborative security architectures across technology stacks, establishing unified trust propagation mechanisms, and ensuring scalability for massive vehicle fleets.

5) Blockchain-based trust mechanisms

Recent research indicates that trust mechanisms empowered by blockchain have become the core technical direction for enhancing the security and trustworthy interaction of the Internet of Vehicles (IoV). Srivastava et al.[56] proposed an Additive Increase and Multiple Decrease (AIMD) trust model based on a permissioned blockchain, which reduces latency while improving the packet delivery rate. Wei et al.[57] proposed a game-theory-driven dynamic Proof of Work (PoW) consensus that adjusts mining difficulty based on RSU trust values; this protocol achieves Nash equilibrium and higher efficiency. Yadav et al.[58] developed a Delegated Proof of Stake (DPoS) consensus that ensures trustworthiness by employing entropy and binomial distribution for miner selection, enabling information traceability and anonymous information sharing. Wang et al.[59] proposed a drone-assisted trust management scheme that combines certificateless authentication and Quality of Service (QoS) evaluation; this scheme resists both internal and external attacks.

Trust mechanisms based on game theory or novel consensus protocols enhance system security. However, these mechanisms typically require complex global information exchange or assume relatively stable network environments. Their effectiveness and efficiency in highly dynamic, partially connected IoV scenarios still require further validation and optimization.

The comparison of related research methods is shown in Table 1.

**Table 1:** Comparison of Related Authentication Schemes.

Authentication Schemes			
Study	Core Method	Advantages	Limitations
Zhong et al. [2]	Registration list-based conditional privacy with ECC	Reduced communication overhead via revocation list replacemen	Centralized maintenance of the registration list may become a performance bottleneck and single point of failure.
Alazzawi et al.[3]	Pseudonym-based, no bilinear pairs/CRL	Efficient signing	The design may not fully ensure unlinkable privacy and is potentially vulnerable to side-channel attacks
Al-Shareeda et al.[4]	Elliptic curve-based conditional privacy	Fixes privacy flaws	Security still primarily relies on a trusted third party, lacking a fully decentralized trust model.
Cui et al. [5]	ECC-based extensible authentication with cloud brokers	Multi-cloud compatibility	Highly dependent on the trusted authority and specific cloud infrastructure, limiting flexibility.
Liu et al. [6]	Dual authentication with reputation evaluation	Dynamic trust assessment; privacy preservation with key agreement	Real-time reputation computation and updates may impose additional performance overhead.
Ma et al.[7]	No bilinear pairs, mutual authentication + session keys	Secure communication	Lacks support for batch verification, which may reduce efficiency in high-concurrency scenarios.
Wu et al. [8]	Certificateless aggregate signatures	Reduces key update frequency	Also lacks batch verification, and the aggregation operation itself may introduce extra computational cost.
Tangade et al.[9]	Identity + HMAC, trust value calculation	Behavior-based trust evaluation	Does not support batch verification of messages and signatures, affecting verification efficiency at scale.
Su et al.[10]	Non-supersingular elliptic curves (V2G networks)	Suitable for V2G scenarios	The scalability and adaptability of the scheme to broader IoV environments are not thoroughly discussed.
Li et al.[11]	Anonymous identity + one-way hash	Identity hiding	The adopted security mechanisms may lead to relatively high computational costs.
Alfadhli et al.[12]	PUF + dynamic pseudonyms	Anti-forgery	Requires integration of specific hardware (PUF), increasing deployment complexity and cost.
Xu et al. [13]	SA/TA/RSU hierarchical management	Clear responsibilities	The core authentication process still relies on a centralized trusted authority, posing a single point of dependency.
Umar et al.[14]	PUF, dynamic pseudonyms	Secure data transfer	Frequent pseudonym updates for anonymity increase system management overhead.
Jiang et al.[15]	PUF + password + biometrics	Multi-factor authentication	The integration of multiple factors increases protocol complexity, implementation, and user experience costs.
Wei et al. [16]	Chebyshev chaotic mapping, fast dynamic grouping	Multi-RSU management	The computational complexity of the chaotic mapping algorithm is not quantified, and its practical performance impact is nclear.
Hou et al.[17]	Lightweight PUF + 5G SIM binding	Prevents identity spoofing	Relies on specific hardware (5G SIM) and carrier environments.
Liu et al.[18]	Certificateless signatures, batch verification	Solves key escrow issues	The use of non-linear pairings may incur relatively high computational overhead.
Xie et al. [19]	PUF + three-factor secrecy	Resists RSU capture attacks	Depends on PUF hardware, and three-factor management increases protocol complexity.
Hou et al. [20]	Blockchain + TEE two-factor auth	Guarantees anonymity, reduces overhead	Deployment of TEE technology relies on specific hardware support and strong security assumptions.
Jiang et al. [21]	Blockchain + ensemble learning + PUF	Two-level security framework	Training and updating the ensemble learning model require significant computational resources and cost.
Xiong et al. [22]	Puncturable signatures + PKI-PRS	Fine-grained forward/backward security	The key management mechanism is complex, posing high requirements for system implementation.
Bhatt et al. [23]	Blockchain-based conditional privacy preservation for temporary platoon communications	Reduces RSU reliance and infrastructure costs; incorporates accident prevention mechanism	Platoon-based trust model may not scale to non-platoon scenarios; blockchain consensus introduces additional latency.
Ibrahim et al. [24]	Six-step authentication process	Preserves privacy, low communication cost	The multi-round interaction in the authentication process may increase communication latency.
Zhang et al. [25]	Platform authentication mechanism	Improves communication security	The description does not specify concrete measures or the strength of privacy protection.
Lo et al.[26]	Edge nodes + consortium blockchain	Decentralized authentication, reduced cloud dependency	Highly reliant on the availability and reliability of edge computing infrastructure.
Mukathe et al.[27]	Schnorr signatures + ECC + reputation incentives	Lightweight, two-message AKA phase	On-chain storage of reputation data may introduce additional blockchain overhead.
Haider et al.[28]	ECC + blockchain PKI management	Low authentication delay & computational cost	Insufficient testing of scalability; performance in large-scale networks needs further validation.
Borges et al.[29]	Self-sovereign identity + MPC-based reputation	Operates without cellular coverage, privacy-preserving	The MPC-based multi-party computation setup is complex and imposes high requirements on participants.
Wang et al.[30]	BCADS: Blockchain-assisted cross-domain authentication with decentralized identity	Enables autonomous identifier management; eliminates single centralized authority; reduces computational cost by 36.6% and communication cost by 87.1%	Consortium blockchain still requires pre-established trust among TAs; regulatory oversight mechanisms add complexity.
Liu et al. [31]	CEDA: Cybertwin-enabled distributed authentication with lightweight cryptography	Real-time behavior tracking; dynamic trust distribution; Edwards-curve-based signature reduces authentication latency	Digital twin maintenance introduces additional computational and storage overhead; performance in ultra-dense scenarios needs further validation.
**Blockchain Schemes**			
**Study**	**Core Method**	**Advantages**	**Limitations**
Dorri et al.[32]	Lightweight blockchain	High scalability	Uses centralized key management lists, posing risks of large-scale key compromise.
Wang et al.[33]	Blockchain + PKI + smart contracts	Integrated solution	The overall scheme has high computational complexity and is not friendly to resource-constrained devices.
Zhang et al. [34]	Blockchain anonymous authentication	Group privacy protection	Lacks sufficient analysis and evaluation of potential communication overhead.
Qureshi et al.[35]	Hyperledger Fabric anonymous sharing	Traceability & unlinkability	Requires a pre-configured blockchain network, limiting deployment flexibility.
Ma et al.[36]	Decentralized automated key management	VANET key lifecycle management	May impose computational and storage pressure on resource-constrained devices in IoV.
Lin et al.[37]	Blockchain + key derivation algorithms	Preemptive certificate management	Does not evaluate the potential additional latency introduced by blockchain consensus and queries.
Li et al.[38]	Blockchain-assisted cross-domain authentication	Supports cross-domain authentication; enables revocation	Group key update overhead; revocation may temporarily affect non-malicious vehicles; additional computational cost
Akhter et al.[39]	Blockchain-based handover authentication	Reduces RSU switching overhead	Does not fully consider the dynamic management challenges posed by high vehicle mobility.
Singh et al.[40]	Lightweight group-based authentication and key agreement for 5G-enabled IoVT	Mutual authentication; forward/backward secrecy; session unlinkability; reduces signaling overhead by 47.3% and bandwidth by 32.3%	5G-specific design limits applicability to non-5G environments; group management overhead in highly dynamic scenarios not fully addressed.
Ali et al.[41]	Bilinear pairing-based signatures	Enhances V2I privacy	Bilinear pairing operations result in low computational efficiency.
Meng et al.[42]	Blockchain-based lightweight cross-regional mutual authentication	Efficient cross-regional node authentication	The authentication process depends on the stability and performance of the underlying blockchain network.
Tan et al.[43]	Edge computing + VANET	Adequate resource provisioning	Effectiveness heavily relies on the coverage density and reliability of edge nodes.
Vishwakarma et al.[44]	Lightweight blockchain + SDN	Secure storage & communication	The temporary credential management mechanism can be complex.
Bhushan et al.[45]	Blockchain miner nodes	Traffic control & security	Does not fully leverage the automation advantages of smart contracts and depends on sensor data quality.
Feng et al. [46]	Asynchronous accumulators, semi-trusted RSUs	Efficient pseudonym management/revocation	The three-tier model structure may introduce additional computational burden.
Xu et al. [47]	Time-sensitive tokens	Low-cost authentication	If not properly designed, the token mechanism could be vulnerable to man-in-the-middle attacks.
Son et al.[48]	Blockchain V2I automation (ECC auth + behavior revocation)	Automated security control	Malicious behavior detection and revocation rely on accurate reporting by RSUs.
Lu et al.[49]	Lattice-based dual blockchain anonymous authentication with forward security and revocability	Quantum-resistant; dual chain decouples identity from mobility; bonsai tree structures ensure forward security; supports anonymous revocation	Lattice-based cryptography incurs higher computational complexity; dual blockchain architecture introduces additional storage and synchronization overhead.
Mei et al.[50]	Blockchain + IoV + edge computing	Efficient computation/storage	Integration of multiple technologies increases overall system complexity.
Xu et al. [51]	Blockchain + RSU-assisted authentication	Solves complex vehicular authentication	Requires well-established RSU infrastructure for support.
Shi et al. [52]	Blockchain DNS + mutual auth	Reduces centralized dependency	Implementation requires support from edge computing facilities.
Surapaneni et al. [53]	Blockchain + IPFS + PoR consensus	Improves re-auth efficiency	The establishment and maintenance of the reputation system incur additional overhead.
Lin et al. [54]	Distributed TTP services	Prevents single-point failure	The distributed consensus mechanism may introduce non-negligible latency.
Ma et al. [55]	Optimized PBFT consensus	Authentication result reuse	PBFT consensus has a clear tolerance limit for Byzantine nodes.
Srivastava et al. [56]	AIMD trust model + permissioned blockchain	Low latency, high packet delivery ratio	Relies on a set of trusted miner nodes.
Wei et al. [57]	Game theory + dynamic PoW + trust evaluation	Nash equilibrium, high consensus efficiency	The quantitative relationship between mining difficulty and trust value is not fully elaborated.
Yadav et al. [58]	Trust-enabled DPoS + entropy/binomial metrics	Traceable anonymous sharing, high detection rate	Trust metric computation may introduce additional computational costs.
Wang et al. [59]	UAV-assisted auth + QoS trust evaluation	Resists insider/outsider attacks, accurate trust assessment	Deployment and maintenance of UAV platforms involve high costs.

Analysis of Table I reveals that existing solutions primarily exhibit three limitations. First, there is an imbalance between security and efficiency, often resulting in high computational overhead for enhanced security or compromised privacy protection for higher efficiency. Second, centralization dependency and scalability issues persist, with trust models or key management presenting single points of failure. Third, deployment complexity and weak dynamic adaptability remain problematic, as many solutions rely on specific hardware or complex cryptography while offering limited support for cross-domain and highly mobile scenarios. Correspondingly, this proposal introduces three core design solutions. First, it employs lightweight cryptographic modules and batch-verifiable anonymous authentication protocols. By integrating security mechanisms such as ECC point multiplication while optimizing efficiency, it achieves a balance between security and performance. Second, it constructs a multi-TA decentralized trust architecture based on blockchain. Through smart contracts, it enables automated trust propagation and key management, eliminating central bottlenecks and enhancing scalability. Finally, it simplifies RSUs to pure relay nodes and designs a cross-domain direct authentication mechanism. This reduces deployment dependencies and leverages the blockchain’s global state to enable rapid, secure mutual recognition among vehicles in dynamic environments. Through this systematic design, the proposed solution comprehensively addresses existing challenges, delivering a more practical and scalable authentication framework for the IoV.

## 4 Security framework and analysis

This section describes the network model of the proposed scheme and its expected security objectives.

### 4.1 System model

**Fig 1:**
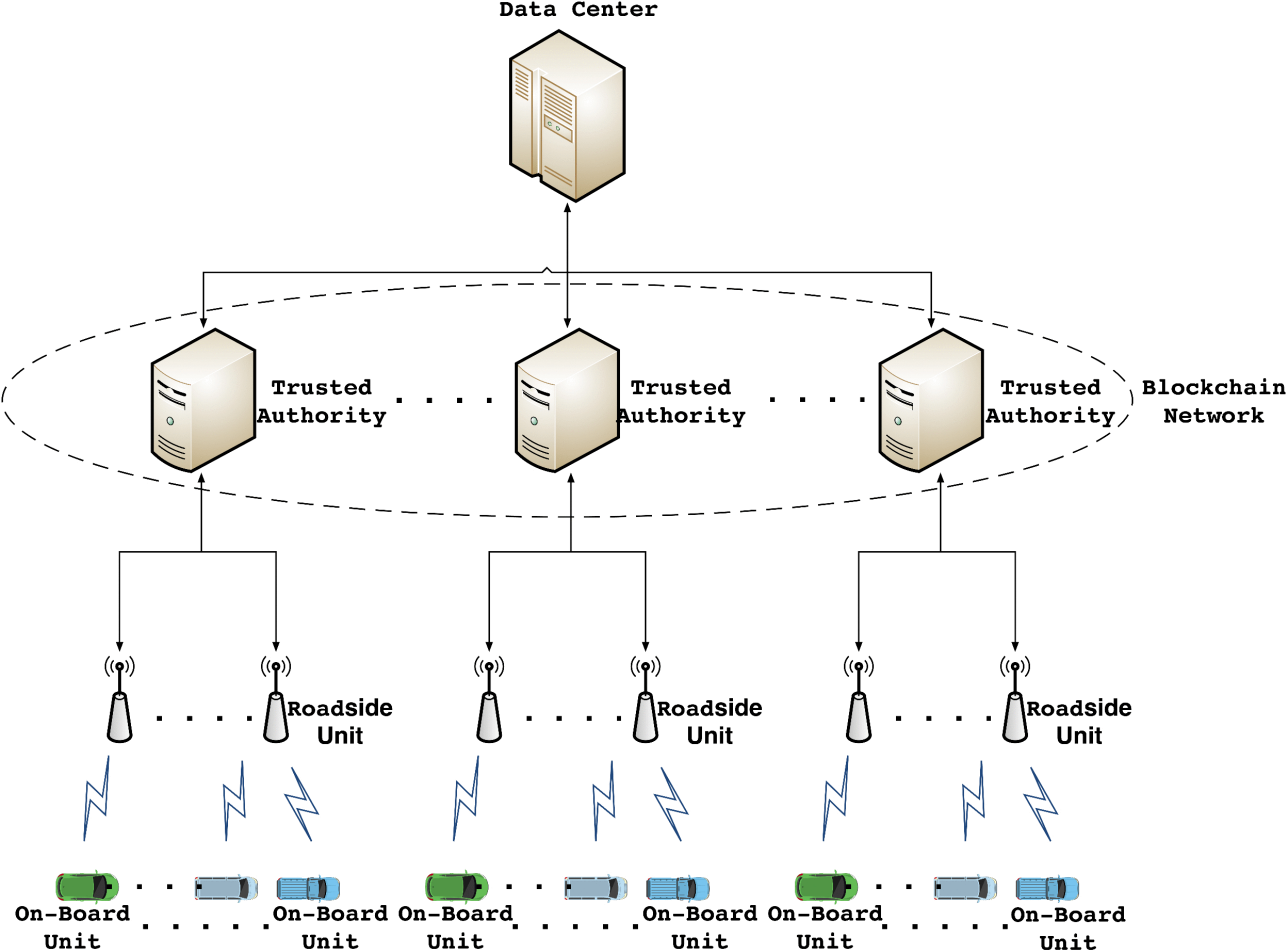
System models. This figure shows that the system model comprises five key components: OBU, RSU, TA, blockchain, and DC.

This section presents the proposed system model, which comprises five key components: On-Board Unit (OBU), Roadside Unit (RSU), Trusted Authority (TA), blockchain, and Data Center (DC). Fig 1 illustrates the system model of the proposed scheme.

OBU: Each vehicle is equipped with an OBU that provides computational and storage capabilities, along with support for cryptographic operations. The primary functions of the OBU include registering with third-party TAs and sending and receiving real-time traffic information via wireless communication with nearby RSUs.

RSU: Installed in road infrastructure, RSUs have limited communication ranges and moderate computational resources. They serve as the first contact point between vehicles and TAs, acting as information bridges. RSUs must register with TAs and facilitate message exchange with vehicles and TAs within their coverage area. Notably, RSUs do not authenticate vehicles or participate in blockchain transactions. It is important to emphasize that complete end-to-end identity authentication and key agreement must always be performed directly between the OBU and the TA. The essential communication exchanges between the OBU and the TA cannot be omitted. Consequently, offloading the authentication function from RSUs does not introduce additional network round-trip delays.

TA: The TA functions as a fully trusted third-party entity with substantial computing and robust storage resources. Both vehicles and RSUs must register with the TA. During vehicle registration, the TA generates and stores shared key parameters on the blockchain for subsequent authentication. When authenticating a vehicle, the TA retrieves the relevant parameters stored on the blockchain. It is worth noting that there are usually multiple TAs in a city or region, and the high-speed, long-distance mobility of vehicles necessitates cross-TA authentication.

Blockchain: Within the proposed network model, the collection of TAs constitutes a private blockchain-based multiserver network, with TAs actively assuming the role of miners. However, it is essential to note that any TA joining the network must be authorized by the system administrator through a meticulous process. Each block of the blockchain contains a pointer to the previous block, through which the corresponding block data can be queried. TAs upload information about registered vehicles to the blockchain, acting as nodes responsible for maintaining the ledger and uploading signed transactions. Regarding the consensus mechanism, to meet the high-speed, low-latency requirements of the IoV, a fast random node selection mechanism based on verifiable random functions (VRF) is adopted (drawing inspiration from Algorand) to participate in block generation. This mechanism efficiently and fairly selects consensus nodes in each round, avoiding the high energy consumption and long delays associated with PoW while eliminating the multi-round communication overhead of Practical Byzantine Fault Tolerance (PBFT). Consequently, it ensures security while enabling rapid and stable block generation.

DC: The DC stores all IoV-related information, including vehicle registration data and encrypted communication records. Importantly, all TAs share a common DC and communicate with it through wired channels. Given that DCs and TAs possess abundant resources, the secure channels established between them ensure message integrity and confidentiality.

In summary, the proposed system model incorporates multiple entities—OBU, RSU, TA, blockchain, and DC—to enable secure communication and authentication in intelligent vehicular networks. The synergy among these entities ensures vehicle security and data integrity.

### 4.2 Threat model

When constructing a secure communication system for the IoV, it is essential to clearly define the capabilities, targets, and methods of potential attackers. This threat model aims to comprehensively analyze the potential security threats, laying a foundation for the subsequent security objectives and measures proposed.

1) Capabilities of potential attackers

Computational Capability: Attackers may possess substantial computational resources, enabling them to perform complex cryptographic analyses, such as breaking keys or forging signatures.

Information Gathering Capability: Attackers may obtain sensitive information by eavesdropping on network communications or infiltrating system nodes, including vehicle identity information and communication keys.

Temporal Flexibility: Attackers can initiate attacks at any time without temporal constraints.

Network Control Capability (Extended from the Dolev-Yao Model): Attackers are assumed to possess complete control over the communication channel, enabling them to intercept, eavesdrop, inject, tamper with, or replay any message transmitted within the network. However, they cannot directly decrypt cryptographically sound encrypted messages without the corresponding keys.

2) Attack targets

Vehicle Identity Information: Attackers may attempt to obtain or tamper with vehicle identity information to conduct impersonation attacks.

Communication Keys: Attackers may attempt to compromise or steal communication keys to eavesdrop on or tamper with communication content.

System Integrity: Attackers may attempt to disrupt the normal operation of the system, for instance, through denial-of-service (DoS) attacks aimed at paralyzing the network.

3) Attack methods

Vehicle Impersonation Attack: Attackers may forge vehicle identities, masquerading as legitimate vehicles to communicate, thereby gaining illegal benefits or compromising system security.

Man-in-the-Middle Attack: Attackers may intercept communications between vehicles and RSUs or TAs, and tamper with or forge communication content to commit fraud or disrupt system operations.

Replay Attack: Attackers may intercept and store legitimate messages, then retransmit them at a later time to deceive the system or vehicles.

Key-Breaking Attack: Attackers may utilize powerful computational resources to attempt to break communication keys to obtain sensitive information or conduct unauthorized communications.

4) Threat model integration and foundational principles

This model systematically integrates core concepts from classical threat modeling frameworks when describing attacker capabilities and methods, ensuring a comprehensive and rigorous analysis:

Dolev-Yao Model: We adopt the Dolev-Yao model’s core assumption regarding attacker network capabilities—namely, attackers fully control communication channels and can perform eavesdropping, interception, injection, and replay operations. This forms the foundation for analyzing network-layer attacks, such as man-in-the-middle and replay attacks, within this model.

STRIDE Model: The attack types addressed by this model span multiple critical dimensions of the STRIDE threat classification:

Spoofing: Corresponds to vehicle impersonation attacks, where adversaries forge identities to masquerade as legitimate entities.

Tampering: In scenarios such as man-in-the-middle attacks, adversaries may illegally modify transmitted data content.

Repudiation: Leveraging blockchain immutability and smart contract execution logs, this proposed solution aims to provide verifiable audit trails for all critical operations to counter such threats.

Information Disclosure: Key cracking attacks and eavesdropping directly threaten information confidentiality.

Denial of Service: Attackers may incapacitate RSUs or TAs through resource exhaustion or similar methods, threatening system availability.

Elevation of Privilege: This solution employs multi-level authentication and strict separation of duties to prevent adversaries from exploiting vulnerabilities to gain unauthorized access beyond their permitted scope.

### 4.3 Security objectives and measures

The proposed scheme addresses the following security requirements through corresponding measures:

1) Registration security: During the registration process, the vehicle must transmit sensitive identity information, which is accomplished over a secure channel to ensure the security of the registration.
2) Confidentiality of TA key: The TA’s master key is neither transmitted over the network nor stored in RSUs at any time to ensure confidentiality.
3) Security of the blockchain pointer: This solution employs a blockchain pointer P as the key index credential for accessing specific on-chain data, such as vehicle identities or transaction records. Unlike the content-addressable approach based on hashes or block heights in conventional blockchains, P undergoes cryptographic processing to provide more direct access control tailored for specific application scenarios. To enhance the pointer’s security, the solution incorporates not only conventional XOR and hash operations but also elliptic curve point multiplication during its computation, thereby strengthening its resistance to cryptographic attacks.
4) Identity anonymity: Identity IDs cannot be directly involved in communication or cryptographic operations to preserve identity anonymity.
5) Communication security between RSU and TA: RSUs do not perform computations—they only act as data relays—thereby mitigating critical data transmission risks between RSUs and TAs.
6) Bidirectional authentication and key agreement: The scheme achieves mutual authentication between the OBU and TA while simultaneously negotiating session keys. Vehicle-to-vehicle communication is enabled via key agreement protocols.
7) Session key strength: To ensure that session keys are not easily cracked, their complexity must be increased.
8) Message integrity: Message integrity is ensured by verifying that the computed hash matches the received hash, confirming the sender’s legitimacy. Hash operations are employed to achieve this property.
9) Forward security: Forward security is ensured by deriving each session key independently using fresh random parameters, such that compromising a current session key does not reveal any previously established session keys.
10) Resistance to common attacks: The solution is designed to resist common attacks, including vehicle impersonation, MITM attacks, and replay attacks.

These security measures collectively ensure that the system remains robust against a diverse range of threats.

## 5 Proposed scheme

This section proposes a blockchain-based anonymous authentication and key agreement scheme for the IoV. The scheme comprises three distinct phases: initialization, registration, and authentication [60]. Table 2 illustrates the notations and descriptions employed in the proposed scheme.

**Table 2:** Summary of Notations and Descriptions Used in the Proposed Scheme.

Notations	Descriptions	Notations	Descriptions
OBU	On-board unit	*G*	Base points on elliptic curves *G*
RSU	Roadside unit	*P*	Blockchain index pointer
TA	Trusted authority	*r*_*i*_	*OBU*_*i*_ ‘s private key
DC	Data center	*r*_*TA*_	TA’s private key
*ID*_*i*_, *ID*_*A*_, *ID*_*B*_	Identity of the *OBU*_*i*_, *OBU*_*A*_, *OBU*_*B*_	*r*_*T*_	Random number generated by TA
*RID*_*j*_	Identity of the sensor node *RSU*_*j*_	*R*_*i*_	*OBU*_*i*_ ‘s public key
*PW*_*i*_	Password of the *OBU*_*i*_	*R*_*TA*_	TA’s public key
*BIO*_*i*_	Biological factors of the *OBU*_*i*_	*·*	Point multiplication operation
*SK*_*O,*_ *SK*_*A*_, *SK*_*B*_	Agreed-upon session key of the *OBU*_*i*_, *OBU*_*A*_, *OBU*_*B*_	*Gen*	Generation process of fuzzy extraction
*SK*_*T*_	Agreed-upon session key of the TA	*Rep*	Recovery process of fuzzy extraction
*K*	Master key of the TA	*α*_*i*_	Random secret generated by fuzzy extraction
‖	Concatenation operator	*β*_*i*_	Auxiliary bit string generated by fuzzy extraction
*T*	Timestamp	*h*(*·*)	Hash function
*ΔT*	Maximum permitted transmission delay	⊕	XOR operator

### 5.1 Initialization phase

The initialization phase is as follows:

Step I1: TA generates *K* and *r*_*TA*_*.*
Step I2: TA initializes List = 0. For every OBU request: List←List+1*.*

### 5.2 Registration phase

The registration phase includes OBU registration and RSU registration.

1) Vehicle registration phase

The process of registering an OBU within any TA involves several steps, which are outlined as follows. During this process, the OBU and TA communicate via a secure channel.

Step R1:OBU registers with TA through a secure channel. The owner inputs *ID*_*i*_*, PW*_*i*_ and *BIO*_*i*_ via the OBU, generates a random number *r*_*i*_ and calculates *R*_*i*_ *= r*_*i*_*·G.* Using biometric fuzzy extraction, it generates *α*_*i*_*, β*_*i*_ by biometric fuzzy extraction, calculates *HID*_*i*_ *= h*(*ID*_*i*_*‖α*_*i*_), *HPW*_*i*_ *= h*(*PW*_*i*_*‖α*_*i*_), *O*_*1*_ *= h*(*ID*_*i*_*‖PW*_*i*_*‖α*_*i*_)⊕*r*_*i*_, and *O*_*2*_ *= h*(*ID*_*i*_*‖PW*_*i*_*‖α*_*i*_*‖r*_*i*_). After completing the above steps, {*HID*_*i*_*,HPW*_*i*_*,R*_*i*_} are sent to TA via a secure channel.

Step R2: TA receives the message and calculates *R*_*TA*_ *= r*_*TA*_*·G*, *R*_*TA-i*_ *= r*_*TA*_*·R*_*i*_, *K*_*TA*_ *= h*(*K‖r*_*TA*_), *OK*_*TA*_ *= K*_*TA*_⊕*h*(*HID*_*i*_*‖HPW*_*i*_), and *THID*_*i*_ *= h*(*HID*_*i*_*‖K*_*TA*_). TA sends {*OK*_*TA*_,*THID*_*i*_,*R*_*TA*_} to the OBU via the secure channel.

Step R3: OBU receives the message, calculates *K*_*TA*_ *= OK*_*TA*_⊕*h*(*HID*_*i*_*‖HPW*_*i*_), and *S*_*1*_ *= r*_*i*_⊕*K*_*TA*_, then sends *S*_*1*_ to TA via the secure channel.

Step R4:TA receives the message, generates blockchain pointer *P*, calculates *r*_*i*_ *= S*_*1*_⊕*K*_*TA*_, *OP = P*⊕*h*(*K*_*TA*_*‖R*_*TA-i*_), *OT = r*_*i*_⊕*h*(*THID*_*i*_*‖K*_*TA*_), and uploads {*HID*_*i*_*,OT,S*_*1*_} to the blockchain block corresponding to the pointer *P*. TA sends {*OP*} to the OBU via the secure channel.

Step R5:OBU receives the message and saves {*OK*_*TA*_,*THID*_*i*_,*O*_*1*_,*O*_*2*_,*OP,r*_*i*_}. The OBU registration is completed.

**Fig 2:**
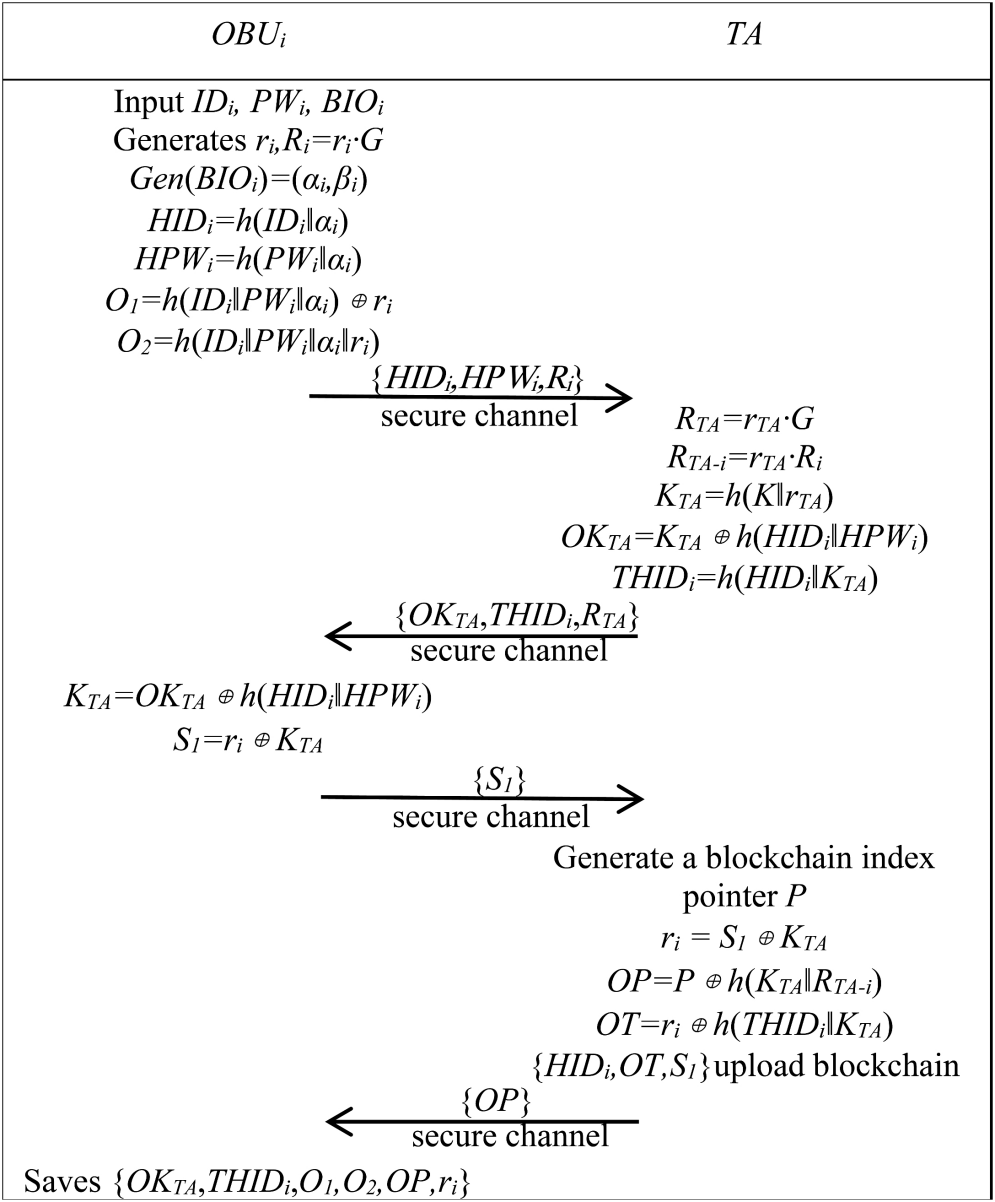
Registration phase between OBU and TA. This figure shows the interaction process when the OBU completes registration with the TA.

Fig 2 illustrates the OBU registration phase.

2) RSU registration phase

Here are the steps for an RSU to register with TA. During this process, the RSU and TA communicate via a secure channel.

Step R1: RSU generates identity information *RID*_*j*_ and a random number *r*_*j*_, then calculates *HRID*_*j*_ *= h*(*RID*_*j*_*‖r*_*j*_) for subsequent authentication. RSU sends {*HRID*_*j*_} to TA via the secure channel.

Step R2: TA receives the message, calculates *TRID*_*j*_ *= h*(*HRID*_*i*_*‖K*_*TA*_), and saves *TRID*_*j*_.

**Fig 3:**
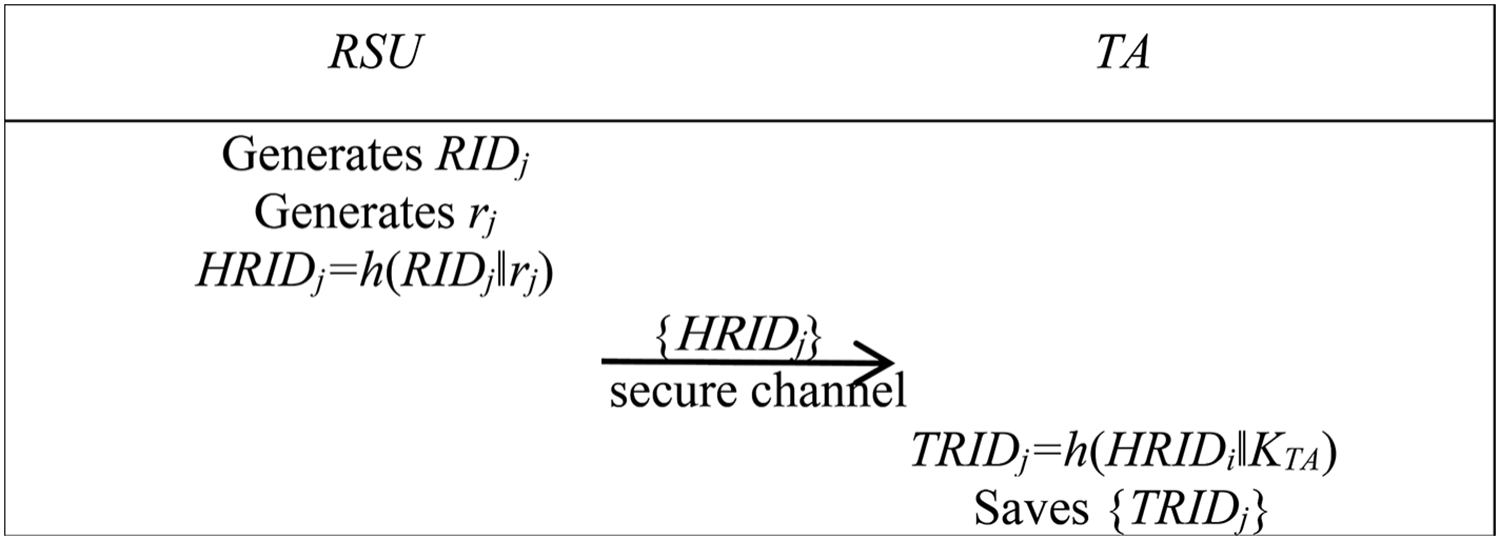
Registration phase between RSU and TA. This figure shows the interaction process when the RSU completes registration with the TA.

Fig 3 illustrates the RSU registration phase.

### 5.3 Authentication and key agreement phase

The authentication phase is divided into two sub-phases: OBU and TA authentication and OBU and OBU authentication. During the subsequent authentication process, all three parties communicate via a public channel.

1) OBU and TA authentication

The authentication and key agreement phase for OBU and TA is as follows.

Step A1: The owner inputs *ID*_*i*_*, PW*_*i*_*, BIO*_*i*_ via OBU, calculates *α*_i_^*^ *= Rep*(*BIO*_*i*_*,β*_*i*_), *r*_i_^*^ = *O*_*1*_⊕*h*(*ID*_*i*_*‖PW*_*i*_*‖α*_i_^*^), *O*_2_^*^ *= h*(*ID*_*i*_*‖PW*_*i*_*‖α*_i_^*^‖r_i_^*^), and checks if *O*_2_^*^ equals *O*_*2*_, If yes, proceed; otherwise, stop. Next, OBU calculates *HID*_*i*_ *= h*(*ID*_*i*_*‖α*_*i*_), *HPW*_*i*_ *= h*(*PW*_*i*_*‖α*_*i*_), *K*_*TA*_ *= OK*_*TA*_⊕*h*(*HID*_*i*_*‖HPW*_*i*_), *OT = r*_*i*_⊕*h*(*THID*_*i*_*‖K*_*TA*_), generates *T*_*1*_, and calculates *M*_*1*_ *= h*(*THID*_*i*_*‖OT‖r*_*i*_*‖K*_*TA*_*‖T*_*1*_). It sends {*M*_*1*_, *OP*, *R*_*i*_, *T*_*1*_} to RSU.

Step A2: RSU receives the message, appends its identity *HRID*_*j*_, and sends {*M*_*1*_, *OP*,*R*_*i*_,*T*_*1*_,*HRID*_*j*_} to TA.

Step A3: TA receives the message and verifies time parameter *T*_*1*_. It then verifies the RSU identity by calculating if *TRID*_j_^*^= h(*HRID*_i_^*^‖K_*TA*_) equals *TRID*_*j*_. If RSU identity is valid, TA calculates *R*_*TA-i*_ *= r*_*TA*_*·R*_*i*_, *P = OP*⊕*h*(*K*_*TA*_*‖R*_*TA-i*_), and checks if blockchain pointer *P* exists. If so, it reads the blockchain data corresponding to {*HID*_*i*_,*OT,S*_*1*_}. Using the read data, it calculates *r*_i_^*^ *= S*_1_^*^⊕*K*_*TA*_, *THID*_*i*_ *= h*(*HID*_i_^*^‖K_*TA*_), OT^*^ *= r*_i_^*^⊕*h*(*THID*_i_^*^‖K_*TA*_), *M*_1_^*^ *= h*(*THID*_i_^*^‖OT^*^‖r_i_^*^‖K_*TA*_*‖T*_*1*_), checks if *M*_1_^*^ equals *M*_*1*_, and if so, performs *List = List+1* to prevent replay attack. TA generates *r*_*s*_, *r*_*T*_ and *T*_*2*_, calculates *r*_*i*_^*’*^ *= h*(*r*_*i*_*‖r*_*s*_), *OT*^*’*^ *= r*_*i*_^*’*^⊕*h*(*THID*_*i*_*‖K*_*TA*_), *R*_*TA-i’*_ *= r*_*TA*_*·r*_*i*_^*’*^*·G*, and derives the session key *SK*_*T*_ *= h*(*THID*_*i*_*‖r*_*i*_*‖r*_*T*_*‖K*_*TA*_*‖R*_*TA-i’*_). It generates a new blockchain pointer *P*^*’*^, calculates *OP*^*’*^ *= P*^*’*^⊕*h*(*K*_*TA*_*‖R*_*TA-i’*_), and puts {*HID*_*i*_*,OT*^*’*^*,SK*} to the new block. Next, it calculates *M*_*2*_ *= r*_*s*_⊕*h*(*HID*_*i*_*‖K*_*TA*_*‖TRID*_*j*_), *M*_*3*_ *= r*_*T*_⊕*h*(*THID*_*i*_*‖K*_*TA*_*‖r*_*s*_), *M*_*4*_ *= h*(*THID*_*i*_*‖TRID*_*j*_*‖r*_*i*_^*’*^*‖r*_*T*_*‖T*_*2*_), *M*_*5*_ *= h*(*THID*_*i*_*‖K*_*TA*_*‖TRID*_*j*_*‖SK*). Finally, {*M*_*2*_,*M*_*3*_,*M*_*4*_, *M*_*5*_, *R*_*TA*_, *T*_*2*_} are sent to RSU.

Step A4: RSU receives the message from TA, appends its identity, and sends {*M*_*2*_,*M*_*3*_,*M*_*4*_*, M*_*5*_*, R*_*TA*_*,HRID*_*j*_*,T*_*2*_} to TA.

Step A5: OBU receives the message and verifies time parameter *T*_*2*_. Then it calculates *TRID*_j_^*^ *= h*(*HRID*_i_^*^‖K_*TA*_), *r*_s_^*^ *= M*_*2*_⊕*h*(*HID*_*i*_*‖K*_*TA*_*‖TRID*_j_^*^), *r*_T_^*^ *= M*_*3*_⊕*h*(*THID*_*i*_*‖K*_*TA*_*‖r*_s_^*^), *r*_*i*_^’*^ *= h*(*r*_*i*_*‖r*_s_^*^), *M*_4_^*^ *= h*(*THID*_*i*_*‖TRID*_*j*_*‖r*_*i*_^’*^‖r_T_^*^‖T_*2*_), and verifies if *M*_4_^*^ equals *M*_*4*_. If verified, it calculates *R*_i-TA_^*^ *= r*_*i*_^’*^·R_*TA*_, derives session key *SK*_O_^*^ *= h*(*THID*_*i*_*‖r*_*i*_*‖r*_T_^*^‖K_*TA*_*‖R*_i-TA_^*^), calculates *M*_5_^*^ *= h*(*THID*_*i*_*‖K*_*TA*_*‖TRID*_j_^*^‖SK^*^), and verifies if *M*_5_^*^ equals *M*_*5.*_ If this also passes, it calculates *O*_*1*_^*’*^ *= h(ID*_*i*_*‖PW*_*i*_*‖α*_*i*_)⊕*r*_*i*_^*’*^, *O*_*2*_^*’*^ *= h*(*ID*_*i*_*‖PW*_*i*_*‖α*_*i*_*‖r*_*i*_^*’*^), and updates OBU’s storage data to {*OK*_*TA*_,*THID*_*i*_,*O*_*1*_^*’*^*,O*_*2*_^*’*^*,OP*^*’*^*,r*_*i*_^*’*^}.

**Fig 4:**
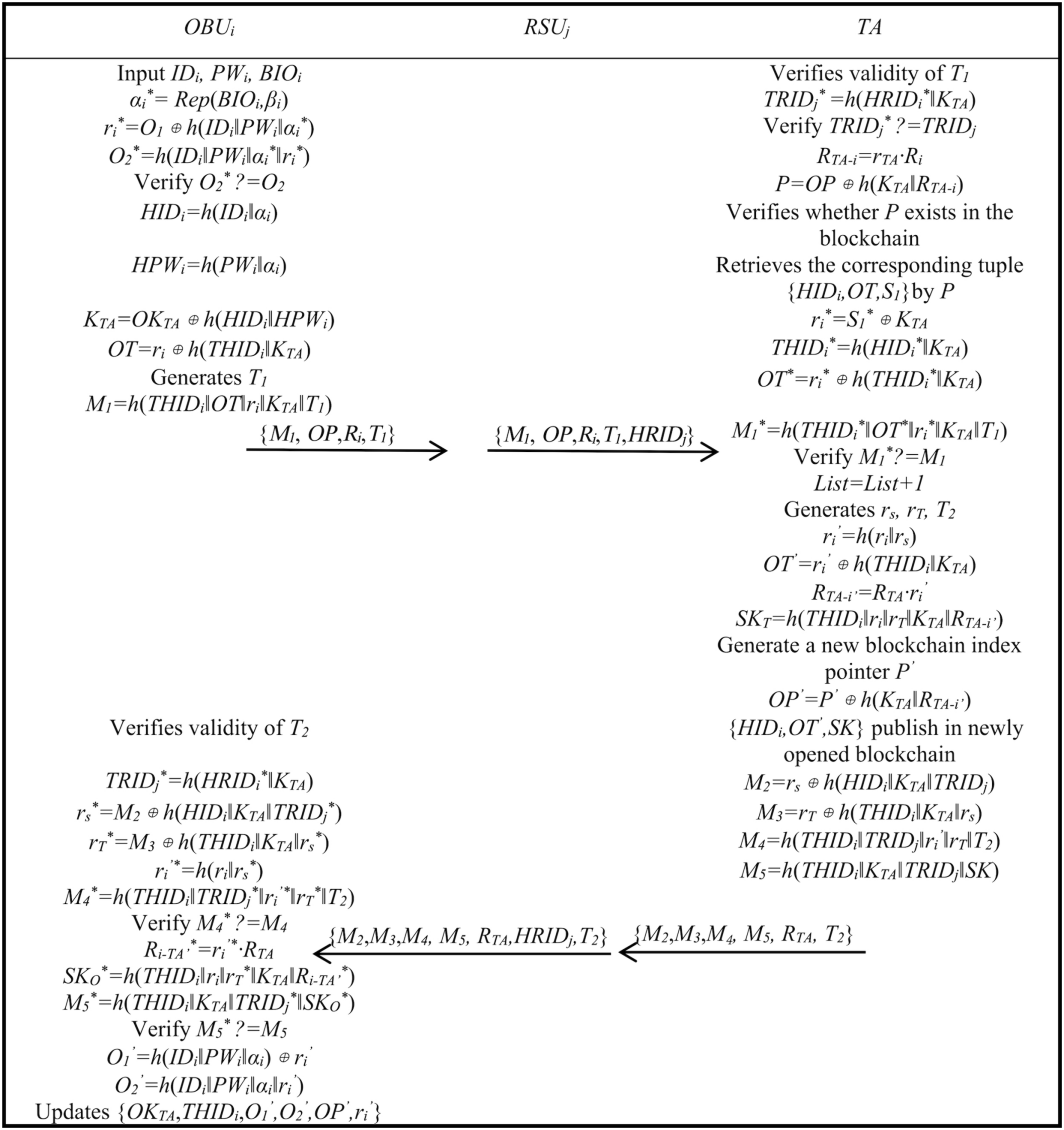
Authentication phase between OBU and TA. This figure shows the process of the OBU completing authentication and key agreement with the TA.

Fig 4 illustrates the authentication and key agreement phase for OBU and TA.

2) OBU and OBU authentication

The authentication and key agreement phase for OBU and OBU is as follows.

Step A1: *OBU*_*A*_ and *OBU*_*B*_ broadcast their respective identity information {*HID*_*A*_,*OP*_*A*_,*R*_*A*_} and {*HID*_*B*_,*OP*_*B*_,*R*_*B*_}. The communicating parties receive each other’s information and send authentication requests to RSU in the region, respectively.

Step A2: RSU forwards authentication requests {*OP*_*A*_,*R*_*A*_,*OP*_*B*_,*R*_*B*_} from *OBU*_*A*_ and *OBU*_*B*_ to TA.

Step A3: TA receives the message and calculates *R*_*TA-A*_ *= r*_*TA*_*·R*_*A*_, *R*_*TA-B*_ *= r*_*TA*_*·R*_*B*_, *P*_*A*_ *= OP*_*A*_⊕*h*(*K*_*TA*_*‖R*_*TA-A*_), *P*_*B*_ *= OP*_*B*_⊕*h*(*K*_*TA*_*‖R*_*TA-B*_). It then checks whether blockchain pointer *P*_*A,*_ and *P*_*B*_ exist in the blockchain. If they do, it reads blockchain data {*HID*_*A*_*,OT*_*A*_*,SK*_*A*_} associated with *P*_*A*_ and {*HID*_*B*_*,OT*_*B*_*,SK*_*B*_} associated with *P*_*B*_, calculates *SK*_*AB*_ *= h*(*SK*_*A*_*‖SK*_*B*_), derives the session keys *PK*_*BA*_ *= SK*_*AB*_⊕*h*(*K*_*TA*_*‖SK*_*B*_) and *PK*_*AB*_ *= SK*_*AB*_⊕*h*(*K*_*TA*_*‖SK*_*A*_) between *OBU*_*A*_ and *OBU*_*B*_, and sends {*HID*_*A*_, *PK*_*BA*_} and {*HID*_*B*_, *PK*_*AB*_} to RSU.

Step A4: RSU sends {*HID*_*A*_, *PK*_*BA*_} to *OBU*_*B*_ and {*HID*_*B*_, *PK*_*AB*_} to *OBU*_*A*_.

Step A5: *OBU*_*B*_ calculates session key *SK*_*AB*_ *= PK*_*BA*_⊕*h*(*K*_*TA*_*‖SK*_*B*_)*, OBU*_*A*_ calculates session key *SK*_*AB*_ *= PK*_*AB*_⊕*h*(*K*_*TA*_*‖SK*_*A*_). *OBU*_*A*_ and *OBU*_*B*_ then communicate using the session key.

**Fig 5:**
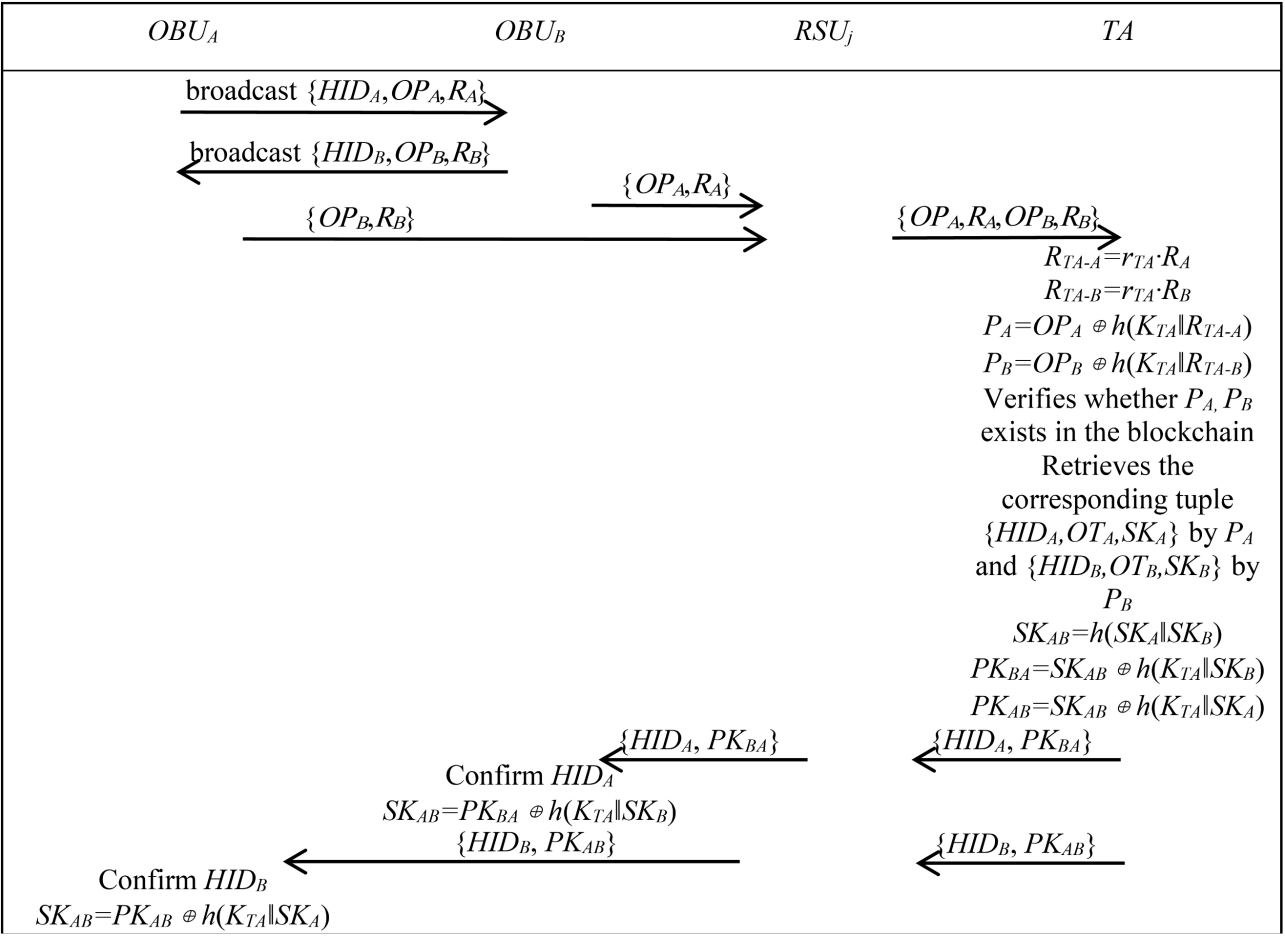
Authentication phase between OBU and OBU. This figure shows the process of authentication and key agreement between two OBUs.

Fig 5 illustrates the authentication and key agreement phase for OBU and OBU.

### 5.4 Scalability analysis and optimization strategy

This scheme focuses on two core bottlenecks in large-scale deployment scenarios, especially in heterogeneous vehicle networks. First, differentiated authentication requirements across multiple vehicle types may lead to an imbalance in TA computational load. With the increasing diversity of vehicles—such as fuel-powered vehicles, new energy vehicles, and autonomous vehicles—authentication dimensions vary significantly. During peak hours, TA computing resources may become overloaded, potentially degrading authentication efficiency. Second, variations in RSU hardware specifications across different regions can cause coordination failures. In heterogeneous networks, discrepancies in RSU processing capabilities may result in mismatched message forwarding rates, with low-capability RSUs becoming data transmission bottlenecks that compromise real-time authentication performance.

To address these challenges, two key optimization strategies are proposed. To tackle the first bottleneck, a dynamic computing power scheduling mechanism is constructed, which sets authentication priorities based on vehicle types and authentication requirements, and diverts non-core authentication tasks to edge nodes. Additionally, a flexible resource pool is configured for the TA, enabling automatic scaling during peak periods to accommodate surges in computational demand. Regarding the second bottleneck, a hierarchical collaborative system for RSUs is established, dividing them into high-computing-power core nodes and ordinary relay nodes based on hardware capabilities. Core nodes are responsible for aggregating and forwarding cross-domain authentication data, whereas ordinary nodes serve only as low-load local relays. By synchronizing RSU load status through blockchain, the system dynamically adjusts data transmission paths to avoid single-point overload and ensure the scalability of the system under large-scale deployment.

## 6 Security analysis

The security of the scheme was assessed using three different security analysis approaches: formal security analysis based on BAN logic [61], informal security analysis to verify security features and resist attacks, and simulation experiments via the ProVerif tool.

### 6.1 Formal security analysis

Next, the scheme’s security was verified using BAN logic, whose notations, descriptions, and inference rules are defined as shown in Tables 3 and 4.

**Table 3:** BAN Logic Notations and Descriptions.

Notations	Descriptions	Notations	Descriptions
P|≡H	*P* believes *H*	$P\overset{SK}{↔}Q$	*P* and *Q* communicate with shared key *SK*.
P⊲H	*P* sees *H*	P|∽H	*P* once said *H*
#(H)	*H* is fresh	P|⇒H	*P* has jurisdiction over *H*
${<H>}_{K}$	*H* encrypted with key *K*		

**Table 4:** Inference Rules of BAN Logic.

Rules	Descriptions	Rules	Descriptions
R1	$\frac{P|\equiv P\overset{SK}{↔}Q,P⊲{<H>}_{SK}}{P|\equiv Q|∽H}$	R4	$\frac{P|\equiv \#(H)}{P|\equiv \#(H,G)}$
R2	$\frac{P|\equiv \#(H),P|\equiv Q|∽H}{P|\equiv Q|\equiv H}$	R5	$\frac{P|\equiv (H,G)}{P|\equiv G}$
R3	$\frac{P|\equiv Q|\equiv H,P|\equiv Q|\Rightarrow H}{P|\equiv H}$	R6	$\frac{P|\equiv \#(H),P|\equiv Q|\equiv H}{P|\equiv P\overset{SK}{↔}Q}$

1) Goals:

$$G\text{1}:TA|\equiv TA\overset{SK}{↔}{OBU}_{i}$$

$$G\text{2}:TA|\equiv {OBU}_{i}|\equiv TA\overset{SK}{↔}{OBU}_{i}$$

$${G\text{3}:OBU}_{i}|\equiv {OBU}_{i}\overset{SK}{↔}TA$$

$${\;G\text{4}:OBU}_{i}|\equiv TA|\equiv {OBU}_{i}\overset{SK}{↔}TA$$

2) Idealized forms:

$${I\text{1}:OBU}_{i}\to {RSU}_{j}:OP\;,R_{i}\;,T_{\text{1}}\;,{<{THID}_{i},{OT,T_{\text{1}}\;,OBU}_{i}|\equiv r_{i}>}_{K_{TA}}$$

$${I\text{2}:RSU}_{j}\to TA:OP\;,R_{i}\;,T_{\text{1}}\;,{HRID}_{j},{<{THID}_{i},{OT,T_{\text{1}}\;,OBU}_{i}|\equiv r_{i}>}_{K_{TA}}$$

$$I\text{3}:TA\to {RSU}_{j}:M_{\text{2}}\;,M_{\text{3}}\;,M_{\text{5}}\;,T_{\text{2}}\;,{<{THID}_{i}\;,{TRID}_{j},T_{\text{2}}\;,TA|\equiv r_{T}>}_{\overset{\text{'}}{\underset{i}{r}}}$$

$${I\text{4}:RSU}_{j}\to {OBU}_{i}:M_{\text{2}}\;,M_{\text{3}}\;,M_{\text{5}}\;,{HRID}_{j},T_{\text{2}}\;,{<{THID}_{i}\;,{TRID}_{j},T_{\text{2}}\;,TA|\equiv r_{T}>}_{\overset{\text{'}}{\underset{i}{r}}}$$

3) Assumptions:

$$A\text{1}:TA|\equiv {OBU}_{i}\overset{K_{TA}}{↔}TA$$

$${A\text{2}:OBU}_{i}|\equiv TA\overset{\overset{\text{'}}{\underset{i}{r}}}{↔}{OBU}_{i}$$

$$A\text{3}:TA|\equiv \#(r_{i})$$

$${A\text{4}:OBU}_{i}|\equiv \#(r_{T})$$

$$A\text{5}:TA|\equiv {OBU}_{i}\;|\Rightarrow (M_{\text{1}})$$

$${A\text{6}:OBU}_{i}|\equiv TA\;|\Rightarrow (M_{\text{4}})$$

$$A\text{7}:TA|\equiv {OBU}_{i}|∽{OBU}_{i}\overset{SK}{↔}TA$$

$${A\text{8}:OBU}_{i}|\equiv TA|∽TA\overset{SK}{↔}{OBU}_{i}$$

4) Main proofs

Step 1: From *I1* and *I2*, we can deduce that *TA* has received message *M*_*1*_.


$$S\text{1}:TA⊲{<M_{\text{1}}>}_{K_{TA}}$$

Step 2: Applying the message-meaning rule (R1) to *S1* and assumption *A1* yields:


$$S\text{2}:TA|\equiv {OBU}_{i}|∽(M_{\text{1}})$$

This confirms that *TA* believes *M*_*1*_ originated from *OBU*_*i*_.

Step 3: By applying the freshness rule (R4) to assumption *A3*, we obtain:


$$S\text{3}:TA|\equiv \#(M_{\text{1}})$$

Thus, *TA* believes that *M*_*1*_ is fresh.

Step 4: Applying the nonce-verification rule (R2) to *S2* and *S3* gives:


$$S\text{4}:TA|\equiv {OBU}_{i}|\equiv (M_{\text{1}})$$

This indicates that *TA* believes *OBU*_*i*_ also believes *M*_*1*_.

Step 5: From *S4* and assumption *A5*, the jurisdiction rule (R3) allows us to infer:


$$S\text{5}:TA|\equiv (M_{\text{1}})$$

Thus, *TA* believes that *M*_*1*_ is true.

Step 6: Applying the conjunction rule (R5) to *S5* and assumption *A5* yields:


$$S\text{6}:TA|\equiv {OBU}_{i}|\equiv r_{i}$$

where *r*_*i*_ is a component of *M*_*1*_. This confirms that *TA* believes that *OBU*_*i*_ also believes *r*_*i*_.

Step 7: With S6, assumption A3, and the session key $SK=h({THID}_{i}\|r_{i}\|r_{T}\|K_{TA}\|R_{TA-i^{\prime }})$, applying rule R6 gives:


$$S\text{7}:TA|\equiv TA\overset{SK}{↔}{OBU}_{i}$$

This achieves goal *G1*, i.e., *TA* believes that *SK* is a secure shared key between itself and *OBU*_*i*_.

Step 8: From *S7*, assumption *A3*, and assumption *A7*, applying rules R2 and R4 yields:


$$S\text{8}:TA|\equiv {OBU}_{i}|\equiv TA\overset{SK}{↔}{OBU}_{i}$$

This achieves goal *G2*, i.e., *TA* believes that *OBU*_*i*_ also believes *SK* is a secure shared key between them.

Step 9: From *I3* and *I4,* we can deduce that *OBU*_*i*_ has received message *M*_*4*_.


$${S\text{9}:OBU}_{i}⊲{<M_{\text{4}}>}_{\overset{\text{'}}{\underset{i}{r}}}$$

Step 10: Applying the message-meaning rule (R1) to *S9* and assumption *A2* yields:


$${S\text{1}\text{0}:OBU}_{i}|\equiv TA|∽(M_{\text{4}})$$

This confirms that *OBU*_*i*_ believes *M*_*4*_ originated from *TA*.

Step 11: By applying the freshness rule (R4) to assumption *A4*, we obtain:


$${S\text{1}\text{1}:OBU}_{i}|\equiv \#(M_{\text{4}})$$

Thus, *OBU*_*i*_ believes that *M*_*4*_ is fresh.

Step 12: Applying the nonce-verification rule (R2) to *S10* and *S11* gives:


$${S\text{1}\text{2}:OBU}_{i}|\equiv TA|\equiv (M_{\text{4}})$$

This indicates that *OBU*_*i*_ believes that *TA* also believes *M*_*4*_.

Step 13: From *S12* and assumption *A6*, the jurisdiction rule (R3) allows us to infer:


$${S\text{1}\text{3}:OBU}_{i}|\equiv (M_{\text{4}})$$

Thus, *OBU*_*i*_ believes that *M*_*4*_ is true.

Step 14: Applying the conjunction rule (R5) to *S13* and assumption *A6* yields:


$${S\text{1}\text{4}:OBU}_{i}|\equiv TA|\equiv r_{T}$$

where *r*_*T*_ is a component of *M*_*4*_. This confirms that *OBU*_*i*_ believes that *TA* also believes *r*_*T*_.

Step 15: With *S14*, assumption *A4*, and the session key $SK=h({THID}_{i}\|r_{i}\|r_{T}\|K_{TA}\|R_{TA-i^{\prime }})$, applying rule R6 gives:


$${S\text{1}\text{5}:OBU}_{i}|\equiv {OBU}_{i}\overset{SK}{↔}TA$$

This achieves goal *G3*, i.e., *OBU*_*i*_ believes that *SK* is a secure shared key between itself and *TA*.

Step 16: From *S15*, assumption *A4*, and assumption *A8*, applying rules R2 and R4 yields:


$${S\text{1}\text{6}:OBU}_{i}|\equiv TA|\equiv {OBU}_{i}\overset{SK}{↔}TA$$

This achieves goal *G4*, i.e., *OBU*_*i*_ believes that *TA* also believes *SK* is a secure shared key between them.

### 6.2 Informal security analysis

Informal security analysis evaluates the scheme’s security properties and its resistance to diverse attacks. Each security property and attack type is briefly defined, followed by a demonstration of how the proposed scheme effectively resists the identified threats.

1) Vehicle anonymity

All vehicle identity information is secured via one-way hashing: the scheme concatenates vehicle *ID* with a random number, then hashes the result to generate *HID*_*i*_ *= h*(*ID*_*i*_*‖α*_*i*_), ensuring that adversaries cannot derive real identities from the hash value.

2) Vehicle impersonation attack

Even if an adversary captures vehicle information and obtains ID/PW, they must input the owner’s biometric *BIO* to verify *α*_*i*_ and compute *r*_i_^*^ = *O*_*1*_⊕*h*(*ID*_*i*_*‖PW*_*i*_*‖α*_i_^*^) and *O*_2_^*^ *= h*(*ID*_*i*_*‖PW*_*i*_*‖α*_i_^*^‖r_i_^*^) for login. The adversary does not possess the biometrics of the vehicle owner, so he cannot log in the system. Furthermore, each login requires a fresh random number *r*_*i*_, that cannot be obtained by the adversary. Therefore, even if the adversary captures the vehicle’s information, he/she cannot execute an impersonation attack.

3) Message integrity

The TA verifies *M*_*1*_ *= h*(*THID*_*i*_*‖OT‖r*_*i*_*‖K*_*TA*_*‖T*_*1*_) upon receiving OBU messages, so if the data are tampered with or deleted, the *M*_*1*_ verification will fail, and the scheme will terminate the verification and subsequent processes. Similarly, the OBU verifies the one-way hash functions *M*_*4*_ *= h*(*THID*_*i*_*‖TRID*_*j*_*‖r*_*i*_^*’*^*‖r*_*T*_*‖T*_*2*_) and *M*_*5*_ *= h*(*THID*_*i*_*‖K*_*TA*_*‖TRID*_*j*_*‖SK*) after receiving the message from the TA, if the data are tampered with or deleted, the verification will fail, thereby ensuring information integrity.

4) Forward safety

Suppose an adversary compromises the vehicle’s current session key *SK*_*O*_ *= h*(*THID*_*i*_*‖r*_*i*_*‖r*_*T*_*‖K*_*TA*_*‖R*_*i-TA*_) through a key attack. Since the key is derived from a hash of parameters (*THID*_*i*_, *r*_*i*_, *r*_*T*_, *K*_*TA*_ and *R*_*i-TA*_), the adversary cannot retrieve these parameters from the hash value alone. Suppose the adversary captures all past data transmitted over the channel to obtain parameters (e.g., *R*_*i*_, *R*_*TA*_), parameters such as *THID*_*i*_, *r*_*i*_, *r*_*T*_, *K*_*TA*_ are never transmitted over the channel, and the random number r_i_ is updated every round. In addition, the attacker cannot calculate *r*_*i*_ and *r*_*TA*_ using *R*_*i*_ *= r*_*i*_*·G* and *R*_*TA*_ *= r*_*TA*_*·G* because this calculation involves the Elliptic Curve Discrete Logarithm Problem (ECDLP) mathematical difficulties. Therefore, an adversary cannot calculate the previous key from the current key.

5) Replay attack

This scheme contains timestamps *T*_*1*_ and *T*_*2*_. During the scheme, when the OBU, RSU, and TA receive a message, they must verify the timestamp. Because the timestamp is generated based on the current time, it is unmodifiable, and the adversary cannot forge it. If the adversary carries out a replay attack, the OBU, RSU, and TA can quickly identify the adversary by recognizing the validity of the timestamp, thereby resisting the replay attack.

6) MITM attack

According to the challenge/response mechanism, both OBU-RSU and RSU-TA communications require mutual identity verification. As established in the analysis of vehicle impersonation attacks, an adversary cannot successfully masquerade as a legitimate entity. Moreover, as demonstrated in the message integrity analysis, an adversary cannot tamper with transmitted messages without detection. Suppose the adversary intercepts the messages {*M*_*1*_, *OP*,*R*_*i*_,*T*_*1*_} and {*M*_*2*_,*M*_*3*_,*M*_*4*_*, M*_*5*_*, R*_*TA*_*, T*_*2*_} over public channels. The messages *M*_*1*_, *M*_*2*_, *M*_*3*_, and *M*_*4*_ are hash functions, and *R*_*i*_ and *R*_*TA*_ are results of elliptic curve scalar multiplications. According to the unidirectional property of hash functions and the mathematical hardness of the ECDLP, the adversary cannot obtain any useful parameters from them. Thus, the adversary cannot disguise themselves as intermediaries during MITM attack. Furthermore, timestamps and random numbers are inherently time-sensitive and cannot be forged via MITM attack, making it impossible for an attacker to pose as an intermediary and initiate an assault.

7) Sybil attack

The proposed scheme mainly includes two measures to resist Sybil attack: binding the vehicle identity with a unique biometric credential, and leveraging the immutability of blockchain. During the registration phase, each OBU needs to complete registration with the TA through exclusive biometric information, which is processed through hashing and stored on the blockchain. During the authentication process, the TA will verify the hash value corresponding to the biometric features against the blockchain records. Due to the tamper-proof nature of blockchain, attackers cannot forge valid biometric credentials. Furthermore, real-time synchronization is achieved through blockchain during multi-TA verification. Even if attackers register false identities in a single TA domain, cross-TA verification can quickly detect anomalies and effectively resist Sybil attacks.

8) DoS attack

This solution employs RSU function optimization and lightweight cryptographic computations to resist DoS attacks. On one hand, RSUs are restricted to functioning solely as message relays, which effectively prevents attackers from exhausting RSU resources through forged authentication requests. On the other hand, the ECC key of this protocol adopts a 160-bit short key length, which not only ensures security but also reduces computation time. Even under high request volumes, the TA can efficiently process legitimate requests and effectively resist DoS attacks targeting system overload.

9) Rogue RSU attack

The dual-layer verification mechanism of this scheme can effectively resist Rogue RSU attack. Legitimate RSUs need to be registered with the TA in advance, and the TA will issue the *OK*_*TA*_ parameter containing the RSU’s identity and public key. Before receiving a message, the OBU and TA will first verify the validity of the operation result corresponding to OK_TA_. Any rogue RSU that fails this verification is immediately rejected. In addition, the TA will send the blockchain pointer parameter OP to legitimate users during registration. Communication between the OBU and TA also requires verification of OP, and rogue RSUs will be directly rejected. Finally, the TA distributes the RSU certificate revocation list to the network through blockchain to ensure that rejected RSUs can be promptly excluded, effectively resisting Rogue RSU attacks.

10) Bidirectional authentication and key agreement

The scheme achieves bidirectional authentication between OBU and TA via *M*_*1*_ *= h*(*THID*_*i*_*‖OT‖r*_*i*_*‖K*_*TA*_*‖T*_*1*_), *M*_*4*_ *= h*(*THID*_*i*_*‖TRID*_*j*_*‖r*_*i*_^*’*^*‖r*_*T*_*‖T*_*2*_), *M*_*5*_ *= h*(*THID*_*i*_*‖K*_*TA*_*‖TRID*_*j*_*‖SK*) and simultaneously derives the session key *SK*_*T*_ *= h*(*THID*_*i*_*‖r*_*i*_*‖r*_*T*_*‖K*_*TA*_*‖R*_*TA-i’*_)= *h*(*THID*_*i*_*‖r*_*i*_*‖r*_*T*_*‖K*_*TA*_*‖R*_*i-TA*_)= *SK*_*O*_.

11) Backward secrecy

The session key *SK* = *h*(*THID*_*i*_*‖r*_*i*_*‖r*_*T*_*‖K*_*TA*_*‖R*_*i-TA*_) in this scheme is jointly generated by the *K*_*TA*_ and the round-specific temporary random numbers *r*_*i*_ and *r*_*T*_. These random numbers are immediately discarded after each session and are never transmitted in plaintext, preventing attackers from obtaining historical *r*_*i*_/*r*_*T*_ values. Consequently, the TA’s master key *K*_*TA*_ alone cannot be used to derive any past *SK*. This ensures strict backward secrecy, protecting all past communication sessions.

12) Session key security

In this scheme, each session key *SK* = *h*(*THID*_*i*_*‖r*_*i*_*‖r*_*T*_*‖K*_*TA*_*‖R*_*i-TA*_)is generated using a unique and independent temporary random number ri and rT for each round. The strong one-way property of the hash function ensures that the random numbers used to generate the key cannot be derived from the old *SK*. More importantly, the random numbers used in different authentication rounds are mathematically and temporally independent. Thus, leakage of random numbers from one session provides no advantage to an adversary in guessing or deriving random numbers for any other session. Therefore, the scheme guarantees session key security.

13) Insider and malicious TA attacks

This solution employs blockchain multi-TA coordination and cryptographic mechanisms to counter insider threats. When a single TA node attempts unauthorized authentication due to key theft or malicious intent, it must provide the OBU’s random number ri for verification by peer TAs. The blockchain’s smart contracts ensure transparent and verifiable trust states. Any abnormal TA behavior is recorded on-chain and excluded through consensus, thereby suppressing internal attacks.

14) Smart-card-lost/device capture attack

The critical login parameter αi in this solution is protected by the vehicle owner’s biometric data (*BIO*_*i*_). The value *O*_*1*_ stored within the device is either a hash or ciphertext. Without *BIO*_*i*_, *α*_*i*_ cannot be recovered nor can valid random numbers *r*_*i*_ be generated. Furthermore, the *r*_*i*_ required for session key generation is dynamically produced each time and is not stored on the device, ensuring physical tampering is ineffective. Therefore, even if the device is lost, an attacker cannot authenticate using the lost device.

Table 5 presents the security characteristics of the proposed scheme alongside a comparative security analysis against various attacks using related schemes from the past two years. In the table, √ denotes protection, and × denotes no protection.

**Table 5:** Comparative Analysis of Security Features and Attack Resilience.

Attacks	Bhatt et al.[23]	Haider et al.[28]	Wang et al.[30]	Li et al.[38]	Proposed scheme
**VA**	×	×	×	×	√
**VIA**	×	√	√	√	√
**MI**	√	√	√	√	√
**FS**	√	√	√	√	√
**RA**	√	×	√	√	√
**MITM**	√	√	×	×	√
**SA**	√	√	√	√	√
**DoS**	√	×	√	√	√
**RRA**	√	√	√	√	√
**BAKA**	√	√	√	√	√
**BCT**	√	√	√	√	√
**BS**	√	√	√	√	√
**SKS**	√	√	√	√	√
**IMTA**	×	×	×	×	√
**SLDCA**	×	×	×	×	√

Note: √ = Supported/Achieved; × = Not Supported/Not Achieved.

Abbreviations: VA = Vehicle Anonymity, VIA = Vehicle Impersonation Attack, MI = Message Integrity, FS = Forward Safety, RA = Replay Attack, MITM = Man-in-the-Middle Attack, SA = Sybil Attack, DoS = Denial-of-Service Attack, RRA = Rogue RSU Attack, BAKA = Bidirectional Authentication and Key Agreement, BCT = Based on Blockchain Technology, BS = Backward Secrecy, SKS = Session Key Security, IMTA = Insider and Malicious TA Attacks, SLDCA = Smart-Card-Lost/Device Capture Attack.

### 6.3 Simulation-based verification using proverif

This section employs the formal verification tool ProVerif to conduct a rigorous security analysis of the proposed authentication and key agreement protocol, thereby validating the security of the scheme. ProVerif, based on the Applied Pi calculus, is an automated verification tool designed to assess confidentiality, authenticity, and integrity in cryptographic protocols. It can analyze a protocol’s resistance to both passive and active attacks.

1) Methodology description

First, model the protocol participants (OBU, RSU, and TA) and their interaction process, including: 1) Defining multiple private channels (sch1, sch2) and public channels (ch1, ch2) for transmitting confidential and public information, respectively; 2) Defining cryptographic operations, including hash functions (h), exclusive-OR operations (xor), point multiplication (mult), symmetric encryption (syme), and biometric-related functions (Gen, Rep); 3) Defining multiple private-type key parameters, such as vehicle identity IDi, temporary key ri, and TA master key K, to prevent theft by attackers.

Second, characterize the authentication process by inserting event markers at critical protocol steps: event beginTA(id) and event endTA(id) mark the start and end of TA authentication; event beginOBUi(id) and event endOBUi(id) mark the start and end of vehicle OBUi authentication.

Finally, verify the protocol’s security properties through the following queries: 1) Using query attacker(SKO) and query attacker(SKT) to conduct confidentiality queries, verifying whether the session keys SKO and SKT have been compromised by attackers; 2) Employing inj-event formal queries to assess bidirectional authentication properties, and conducting authentication queries to verify whether communication between TA and OBUi is susceptible to identity spoofing or replay attacks.

2) Analysis process

The entire system analysis process is divided into three parallel processes:!processOBUi |!processRSU |!processTA, representing the behaviors of the vehicle OBU, RSU, and TA respectively. These include: 1) OBUi uses biometric information Bio to generate auxiliary data (alpha_i, beta_i), calculates the hidden identity HIDi and hidden password HPWi, and sends registration-related data to TA; 2) The TA uses the master key K and random number rTA to generate key material, returning data such as OKTA, temporary identity THIDi, and public key parameters RTAA to OBUi to complete OBUi registration; 3) OBUi and TA complete mutual authentication through multiple interactions, ultimately negotiating session keys SKO and SKT. RSU acts as a relay node, forwarding authentication messages between OBUi and TA without participating in key derivation or security parameter processing.

During verification, ProVerif automatically simulates the Dolev-Yao attacker model. Under this model, the attacker fully controls the public channel, capable of eavesdropping, intercepting, tampering with, and injecting messages, while possessing cryptographic computation capabilities. ProVerif detects whether the attacker can violate the defined security properties by exploring attack paths in the state space and deriving rules.

3) Analysis results

**Fig 6:**
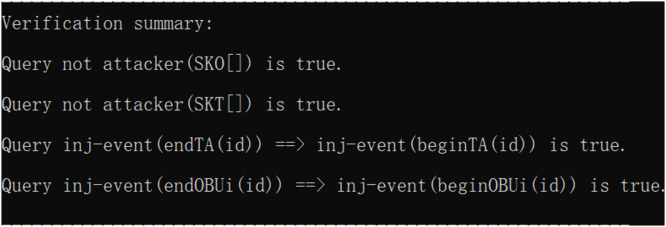
Outputs of the proVerif verification. This figure shows the automated verification results generated by ProVerif.

The automated verification results from ProVerif are shown in Fig 6. Based on the verification results, the protocol’s security is analyzed as follows.

Session key confidentiality is strictly guaranteed. Verification results (1) and (2) show that both “not attacker(SKO[])” and “not attacker(SKT[])” yield true. This indicates that even under the Dolev-Yao threat model, where the attacker fully controls the public channel and employs eavesdropping, replay, or tampering attacks—the attacker cannot obtain the session keys SKO and SKT negotiated between the vehicle and the TA. These results validate the security of the protocol’s key exchange process, thereby ensuring the confidentiality of subsequent communications.

Furthermore, the bidirectional authentication mechanism is effectively implemented. Verification results (3) and (4) demonstrate that “inj-event(endTA(id)) ==> inj-event(beginTA(id))” and “inj-event(endOBUi(id)) ==> inj-event(beginOBUi(id))” both yield true. This confirms the protocol’s capability to achieve secure mutual authentication. Specifically, only after the TA has indeed initiated an authentication session with a specific vehicle OBUi (beginTA(id)) can that session successfully conclude (endTA(id)); similarly, only after the vehicle OBUi has correctly initiated the authentication process (beginOBUi(id)) can it successfully complete authentication with the TA (endOBUi(id)). This causal relationship between injection events (inj-event) proves the protocol’s resistance to MITM attack and identity spoofing attack, ensuring the authenticity of both communicating parties.

## 7 Performance analysis and comparison

To evaluate the performance of the proposed scheme, two key metrics are introduced: computational cost and communication cost. These metrics are calculated and compared with those of existing schemes. Here, computational cost refers to the time resources expended in executing the necessary processing tasks, and communication cost denotes the quantified volume of data bits transmitted through the communication channel.

### 7.1 Computational cost

The computational cost is defined as the total simulation time required to fully execute the proposed scheme, including operations such as authentication, verification, and key agreement. This study focuses on elliptic curve operations, modular exponentiation, symmetric cryptography, and hashing. XOR and concatenation costs are negligible compared to others. In this section, the execution time data for various cryptographic primitives are examined, employing the well-regarded Multi-precision Integer and Rational Arithmetic Cryptographic Library (MIRACL) as the benchmark, in accordance with established literature [62]. The considered hardware configuration has the setting: “Raspberry PI 3 B+ Rev 1.3, Ubuntu 20.04 LTS, 64-bit OS, 1.4 GHz Quad-core processor, cores 4, 1 GB RAM”. The measured computation times for the relevant cryptographic operations are presented in Table 6.

**Table 6:** Computation Time Measurements for Cryptographic Operations.

Notations	Descriptions	Time(ms)	Notations	Descriptions	Time(ms)
*T*_*h*_	one-way hash function using SHA-256 hashing algorithm	0.309	*T*_*bp*_	bilinear pairing operation	32.084
*T*_*exp*_	modular exponentiation	0.228	*T*_*sm-bp*_	bilinear pairing point multiplication	13.313
*T*_*ecm*_	elliptic curve point (scalar) multiplication	2.288	*T*_*mtp*_	point-to-map function	0.385
*T*_*eca*_	elliptic curve point addition	0.016	*T*_*ecenc*_	ECC encryption	4.592
*T*_*senc*_ */T*_*sdec*_	symmetric key (Advanced Encryption Standard AES-128) encryption/decryption	0.018	*T*_*ecdec*_	ECC decryption	2.304
*T*_*mul*_	modular multiplication	0.011	*T*_*sig*_	signature operation	13.405
*T*_*add*_	modular addition	0.010	*T*_*ver*_	verification operation	4.33

Assuming that the computational costs of *OBU*_*i*_, RSU, and TA are *ECA*_*OBUi*_, *ECA*_*RSU*_, and *ECA*_*TA*_, respectively, the specific computational expressions are as follows:


$${ECA}_{OBUi}={\text{1}T}_{ecm}+{\text{21}T}_{h}$$ (1)

$${ECA}_{RSU}={\text{1}T}_{h}$$ (2)

$${ECA}_{TA}={\text{3}T}_{ecm}+{\text{17}T}_{h}$$ (3)

The total computation costs *ECA*_*total*_ of the scheme are as follows:


$${ECA}_{total}={\text{4}T}_{ecm}+{\text{39}T}_{h}$$ (4)

**Fig 7:**
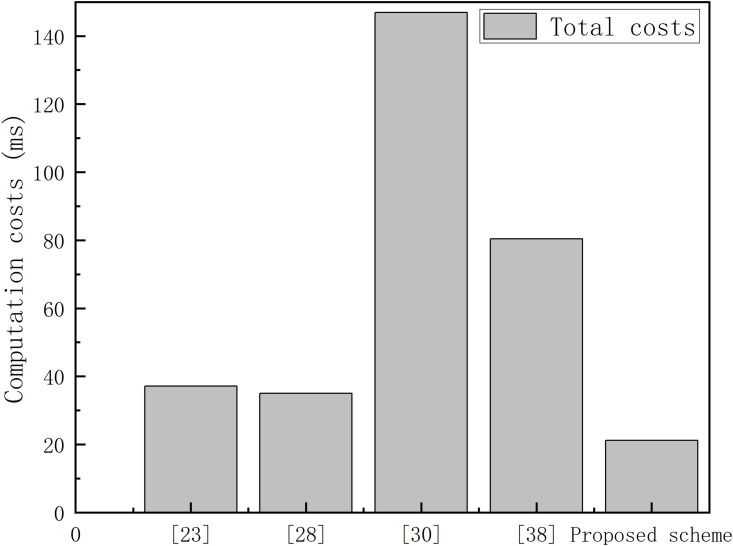
Comparison of computation costs of various schemes. The figure illustrates the comparison of the computation costs of various schemes.

To substantiate the efficacy of the proposed scheme and validate its performance, its computational costs were compared with those of related schemes from the past two years. Fig 7 illustrates this comparison. The proposed scheme achieves the lowest computational cost among all compared solutions. It employs an ECDH-based key agreement mechanism, where the majority of the computational overhead is attributed to point multiplication operations. Although point multiplication is relatively time-intensive, it offers higher key density and enables the use of shorter key lengths while maintaining an equivalent security level. Consequently, despite incorporating this operation, the overall computational cost of the proposed scheme does not increase significantly, thereby effectively enhancing the operational efficiency of the IoV system.

### 7.2 Communication cost

Communication cost refers to the bits required to transmit variables and parameters. This study evaluates costs based on data lengths, including hash values, timestamps, and elliptic curve points (public keys). For consistency with other compared schemes, the proposed scheme adopts the data length settings defined in reference [30]. The specific values are detailed in Table 7.

**Table 7:** Communication Data Length Analysis for the Proposed Scheme.

Notations	Descriptions	Length(bit)
*L*_*h*_	Length of hash value	256
*L*_*T*_	Length of timestamp	64
*L*_*ECC*_	Length of points of elliptic curve (public key)	1024

Assuming that the length of the data sent by *OBU*_*i*_, RSU, and TA is *ECO*_*OBUi*_, *ECO*_*RSU*_, and *ECO*_*TA*_, respectively, the specific calculation expression is as follows:


$${ECO}_{OBUi}=L_{ECC}+{\text{2}L}_{h}+{\text{1}L}_{T}$$ (5)

$${ECO}_{RSU}={\text{2}L}_{ECC}+{\text{8}L}_{h}+{\text{2}L}_{T}$$ (6)

$${ECO}_{TA}=L_{ECC}+{\text{4}L}_{h}+{\text{1}L}_{T}$$ (7)

The total transmit data length *ECO*_*total*_ is as follows:


$${ECO}_{total}={\text{4}L}_{ECC}+{\text{14}L}_{h}+{\text{4}L}_{T}$$ (8)

**Fig 8:**
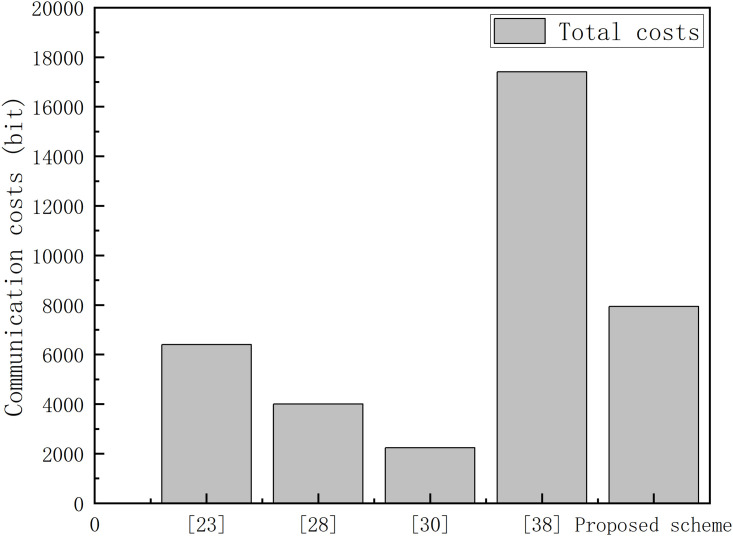
Comparison of communication costs of various schemes. The figure illustrates the specific quantification of the communication costs of various schemes.

To assess the effectiveness of the proposed scheme, a comprehensive comparison of its communication costs with those of the aforementioned schemes was conducted. Fig 8 presents a quantitative comparison of communication costs across different schemes. As illustrated, the communication overhead of the proposed scheme falls within the mid-to-high range among all compared solutions. This result is primarily attributed to the transmission of multiple public keys and hash values during the authentication process, which enhances security and attack resistance by incorporating redundant security information. Although this leads to a slight increase in communication overhead, the overall cost remains within a reasonable and controllable range acceptable for practical IoV deployments. Thus, the proposed scheme achieves a balanced trade-off between security and communication efficiency.

## 8 Conclusion

This study proposes a blockchain-based scheme for anonymous authentication and key agreement in IoV, leveraging a multi-TA model and blockchain architecture. With the help of the blockchain, vehicle OBUs, RSUs, and TAs are integrated and authenticated, and secure storage is provided. The TAs in this scheme form a blockchain network that solves the problem of cross-TA vehicle authentication. Security is ensured via bidirectional authentication and key agreement between OBUs and TAs. Formal security verification using BAN logic demonstrates the scheme’s robustness against various attacks, while simulation analysis with the ProVerif tool confirms its practical feasibility. Performance evaluation of computational and communication overhead indicates that the proposed scheme achieves lower costs compared to most existing protocols. Although the proposed solution achieves a favorable balance between security and performance, it still exhibits certain limitations. Future work will focus on optimizing the following two aspects. First, the current reliance on elliptic curve cryptography results in relatively high computational overhead. Subsequent research will explore lightweight cryptographic algorithms or hardware acceleration techniques to improve efficiency. Second, cross-TA online collaborative authentication is sensitive to network latency. We plan to design mechanisms that support local verification and asynchronous consensus to enhance system robustness. Overall, this study provides a viable pathway toward achieving secure and efficient cross-domain authentication for IoV, and the proposed optimizations will further advance research in this area.
